# *CTB6* Confers Cold Tolerance at the Booting Stage by Maintaining Tapetum Development in Rice

**DOI:** 10.1002/advs.202411357

**Published:** 2025-01-22

**Authors:** Shilei Gao, Jin Li, Yawen Zeng, Huahui Li, Zhenhua Guo, Haifeng Guo, Meng Zhang, Yunsong Gu, Runbin Su, Wei Ye, Andong Zou, Xingming Sun, Zhanying Zhang, Hongliang Zhang, Yongmei Guo, Wendong Ma, Pingrong Yuan, Zichao Li, Jinjie Li

**Affiliations:** ^1^ Frontiers Science Center for Molecular Design Breeding, Beijing Key Laboratory of Crop Genetic Improvement, College of Agronomy and Biotechnology China Agricultural University Beijing 100193 China; ^2^ Biotechnology and Germplasm Resources Institute Yunnan Academy of Agricultural Sciences Kunming 650205 China; ^3^ Institute of Food Crop Research Yunnan Academy of Agricultural Sciences Kunming 650205 China; ^4^ Rice Research Institute Heilongjiang Academy of Agricultural Sciences Jiamusi 154026 China

**Keywords:** cold tolerance, CTB6, pollen fertility, tapetum development, the booting stage

## Abstract

Rice is highly sensitive to cold stress, particularly at the booting stage, which significantly threatens rice production. In this study, we cloned a gene, *CTB6*, encoding a lipid transfer protein involved in cold tolerance at the booting stage in rice, based on our previous fine‐mapped quantitative trait locus (QTL) *qCTB10‐2*. *CTB6* is mainly expressed in the tapetum and young microspores of the anther. CTB6 interacts with catalases (CATs) to maintain their stability, thereby scavenging reactive oxygen species (ROS) accumulation in anthers and facilitating tapetum development under cold stress conditions. Additionally, CTB6 has lipid‐binding ability and affects the lipid content in anthers to regulate cold tolerance at the booting stage. Haplotype analysis and promoter activity assay revealed a specific single nucleotide polymorphism (SNP)‐489 variation in the promoter of *CTB6*, which enhances its expression and results in improved cold tolerance in Hap1‐K varieties. The *CTB6* near‐isogenic line (NIL) exhibited enhanced cold tolerance at the booting stage, with no significant effects on other agronomic traits. Our findings uncover a natural variation of *CTB6* for cold tolerance at the booting stage and provide new genetic resources for cold tolerance breeding in rice.

## Graphical abstract

In this study, we cloned a novel cold‐tolerance gene, *CTB6*, from the Yunnan plateau cultivar KMXBG. *CTB6* is mainly expressed in the tapetum and young microspores of anthers. It regulates tapetum development by maintaining the stability of catalases and increases the lipid content in anthers to enhance the cold tolerance at the booting stage in rice.

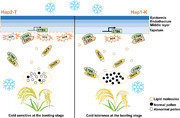

## 1 Introduction

Rice, as a tropical and subtropical crop, is sensitive to low temperature,^[^ ^1^ ^]^ which is a major environmental factor restricting rice growth and development.^[^ ^2^ ^]^ Cold stress at the booting stage of rice leads to a direct reduction in seed‐setting rate and a decrease in yield. Cold tolerance is a complex quantitative trait controlled by multiple genes and is easily influenced by the environment.^[^ ^3^ ^]^ Therefore, few cold‐tolerance genes at the booting stage have been identified through forward genetic approaches. *Ctb1* encodes an F‐box protein, which may be involved in cold tolerance at the booting stage via the ubiquitin‐proteasome pathway.^[^ ^4^ ^]^ *CTB4a* encodes a leucine‐rich repeat‐like receptor kinase that interacts with Atp β to regulate the energy supply by mediating ATP levels under cold stress conditions.^[^ ^3^ ^]^ *LTT1* participates in cold tolerance at the booting stage by maintaining reactive oxygen species (ROS) homeostasis in anthers under cold stress conditions.^[^ ^5^ ^]^ *CTB2* encodes a UDP‐glucose sterol glucosyltransferase, enhancing cold tolerance at the booting stage via the sterol pathway.^[^ ^6^ ^]^ *LEA9* is a newly discovered cold tolerance gene at the booting stage identified through a genome‐wide association study (GWAS). *LEA9* encodes a late embryogenesis abundant protein, but its mechanism in cold tolerance needs further elucidation.^[^ ^7^ ^]^ As indicated above, the mechanisms of cold tolerance at the booting stage remain unclear, and the cold‐tolerance genes for rice breeding require further exploration.

Lipid transfer proteins (LTPs) are small and basic proteins that are commonly present in plant species. The N‐terminus of LTPs contains a signal peptide, and the LTP domain includes eight conserved cysteine residues.^[^ ^8^ ^]^ In rice, several LTPs are reported to regulate lipid transport and play an important role in the development of anthers and pollen grains. *OsC6* encodes an LTP that is highly expressed in the tapetum and microspores, participates in Ubisch body development, and transports lipid molecules from the tapetum to pollen and the anther exine.^[^ ^9^ ^]^ *EPAD1*, also known as *OsLTPL94*, is expressed in pollen mother cells and young microspores.^[^ ^10^ ^]^ EPAD1 can bind to phospholipid molecules, potentially participating in the development of microspore germination pores.^[^ ^11^ ^]^ OsLTPL47 possesses lipid‐binding ability and interacts with OsC6, which may promote the transport of lipid to the pollen exine, contributing to pollen development in a relay manner.^[^ ^12^ ^]^ Additionally, LTPs were reported to play significant roles in various stress responses, growth, and development processes, such as salt, cold, drought and disease resistance, as well as seed development.^[^ ^13^ ^]^ In chrysanthemums, DgTIL1 interacts with DgLTP to enhance the stability of DgLTP, which may confer cold tolerance through the ROS scavenging pathway.^[^ ^14^ ^]^ Although LTPs play a crucial role in regulating lipid distribution, transport, and maintaining cell membrane integrity, there have been no reports on LTPs being involved in cold tolerance at the booting stage in rice, and the underlying molecular mechanism remains unclear so far.

ROS serve as signaling molecules for tapetum degradation.^[^ ^15^ ^]^ In *Arabidopsis*, *RBOHE* participates in the programmed cell death (PCD) of the tapetum by regulating ROS levels.^[^ ^15^ ^]^ In rice, WA352 interacts with COX11 to inhibit its ROS scavenging ability, leading to premature PCD of the tapetum and pollen sterility.^[^ ^16^ ^]^ Similarly, DTC1 interacts with MT‐2b to inhibit its ROS scavenging ability, resulting in delayed tapetum degradation in *dtc1* mutant plants.^[^ ^17^ ^]^ *EDT1* encodes a subunit of ATP citrate lyase; in *edt1* mutant plants, tapetum degradation is accelerated due to increased ROS levels and reduced energy load.^[^ ^18^ ^]^ Additionally, *LTT1* is a mutant with enhanced cold tolerance at the booting stage; *ltt1* was found to increase ROS accumulation in the corresponding wild‐type anthers, which accelerated the degradation of the tapetum and ultimately led to pollen sterility.^[^ ^5^ ^]^ Therefore, tapetum development can be achieved by modulating ROS levels, with excessive or insufficient ROS can lead to abnormal tapetum degradation, ultimately resulting in abnormal pollen grain development.

In this study, we identified the cold‐tolerance gene, *CTB6*, for cold tolerance at the booting stage based on a previously fine‐mapped quantitative trait locus (QTL) *qCTB10‐2*. *CTB6* encodes an LTP and interacts with catalases (CATs), maintaining their stability under cold stress to scavenge abnormal ROS in anthers. Moreover, CTB6 has lipid‐binding ability and enhances cold tolerance at the booting stage by increasing the lipid content in anthers. The SNP‐489 variation in the promoter of *CTB6* enhances its expression, thereby improving cold tolerance at the booting stage. *CTB6* is under selection in temperate *japonica* rice, potentially facilitating adaptation to the cold climate of the Yunnan‐Guizhou Plateau. The near‐isogenic line (NIL) of *CTB6* showed a higher seed‐setting rate without affecting other traits under cold stress conditions. Our findings provide a potential genetic locus for improving cold tolerance in *japonica* rice.

## 2 Results

### 2.1 *CTB6* Positively Regulates Cold Tolerance at the Booting Stage

In our previous study, a QTL, *qCTB10‐2*, was identified for cold tolerance at the booting stage.^[^ ^19^ ^]^ We generated a NIL containing *qCTB10‐2* from a cross between the cold‐tolerant Yunnan cultivar KUNMINGXIAOBAIGU (KMXBG) and the cold‐sensitive Japanese cultivar Towada (Figure S1, Supporting Information). To identify the candidate gene of *qCTB10‐2*, we performed sequencing analysis of candidate genes between KMXBG (K) and Towada (T), revealing the presence of single nucleotide polymorphisms (SNPs) in the promoter and coding regions (CDS) of *Os10g11770*, *Os10g11810*, *Os10g11750* and *Os10g11730* (Table S1, Supporting Information). Since the expression of *Os10g11770* is very low in anthers and difficult to detect, we knocked out *Os10g11810*, *Os10g11750* and *Os10g11730* in the NIL background (Figures S2A,S3A,S4A, Supporting Information). Owing to the absence of significant differences in seed‐setting rates between the *Os10g11810* knockout lines and the NIL under cold stress in a high‐altitude area (CS‐HAA) (Figure S2B–D, Supporting Information), we suggested that *Os10g11810* was not the candidate gene for cold tolerance at the booting stage. Besides *Os10g11750* knockout lines, we generated two types of *Os10g11750* CDS overexpression lines (Figure S3D, Supporting Information). The result of phenotypic evaluation showed that the seed‐setting rates of both *Os10g11750* ^K^ overexpression and knockout lines were significantly lower than their wild types under CS‐HAA (Figure S3B,C,E,F Supporting Information), suggesting that *Os10g11750* is likely not the candidate gene for cold tolerance at the booting stage.

**Figure 1:**
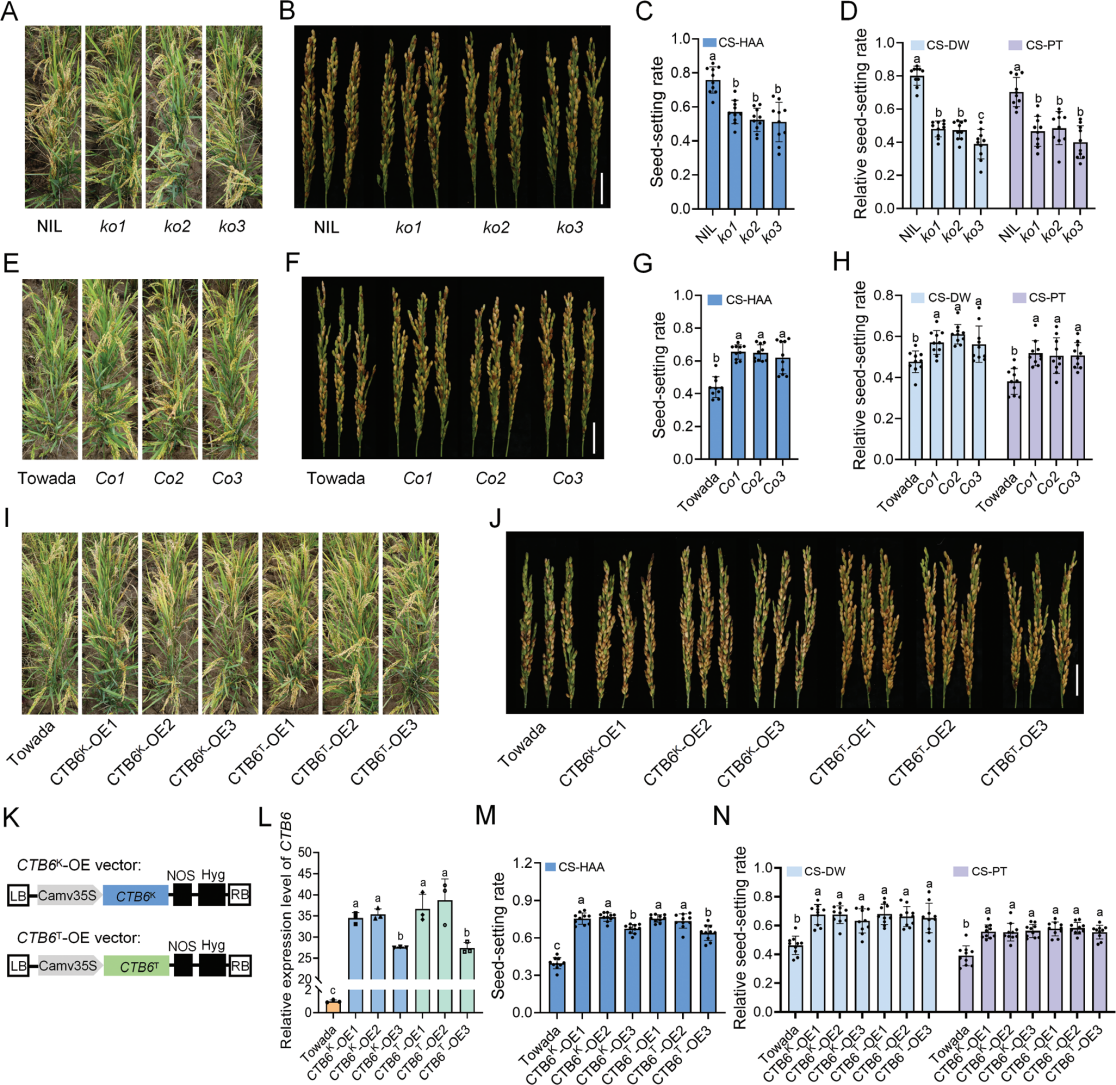
Functional analysis of *CTB6* in response to cold stress at the booting stage. A,B) Phenotype of plants A) and panicles B) of NIL and *CTB6* knockout lines grown under CS‐HAA. Scale bar = 2 cm. C) Seed‐setting rates of NIL and *CTB6* knockout lines under CS‐HAA. D) Relative seed‐setting rates of NIL and *CTB6* knockout lines under CS‐DW and CS‐PT. E,F) The phenotype of plants E) and panicles F) of *CTB6* complementation lines grown under CS‐HAA. Scale bar = 2 cm. G) Seed‐setting rates of Towada and *CTB6* complementation lines under CS‐HAA. H) Relative seed‐setting rates of Towada and *CTB6* complementation lines under CS‐DW and CS‐PT. I,J) The phenotype of plants I) and panicles J) of *CTB6* overexpression lines grown under CS‐HAA. Scale bar = 2 cm. K) Schematic of vectors for transgenic analysis. *CTB6* ^K^‐OE, *CTB6* ^KMXBG^ overexpression vector; *CTB6* ^T^‐OE, *CTB6* ^Towada^ overexpression vector. L) Overexpression efficiency of *CTB6* in transgenic lines by RT‐qPCR. *Actin1* was used as an internal reference. Data are means ± SD (*n* = 3), and the significance of the difference was calculated with a one‐way ANOVA analysis–Duncan test. M) Seed‐setting rates of Towada and *CTB6* overexpression lines under CS‐HAA. N) Relative seed‐setting rates of Towada and *CTB6* overexpression lines under CS‐DW and CS‐PT. CS‐HAA, cold stress in high‐altitude areas; CS‐DW, cold stress in deep water; CS‐PT, cold stress in a phytotron. In C, D, G, H, M, and N, the data are means ± SD (*n* = 10), and the significance of the differences was calculated with a one‐way ANOVA analysis–Duncan test.

We phenotypically evaluated the *Os10g11730* knockout lines under CS‐HAA, which showed significantly lower seed‐setting rates compared with the NIL (**Figure** **1**A–C). Additionally, phenotypic identification through deep cold water irrigation (CS‐DW) and phytotron treatment (CS‐PT) revealed significantly lower relative seed‐setting rates of the *Os10g11730* knockout lines compared with the NIL (Figure 1D). To further validate the function of *Os10g11730* in cold tolerance at the booting stage, we complemented the *Os10g11730* gene from KMXBG into Towada (Figure S4B, Supporting Information). The complemented lines exhibited significantly higher seed‐setting rates than Towada under all three cold stress conditions (Figure 1E–H). Therefore, we demonstrated that *Os10g11730* played an important role in regulating cold tolerance at the booting stage and named it *CTB6*.

*CTB6* encodes an LTP consisting of 166 amino acids. A phylogenetic tree construction showed that CTB6 and its homologs are present only in grasses (Figure S5A,B and Table S2, Supporting Information). Owing to that the three SNPs in the coding regions of *CTB6* result in two non‐synonymous mutations (Figure S5C, Supporting Information), we conducted subcellular localization studies for CTB6^K^‐GFP and CTB6^T^‐GFP. Both CTB6^K^‐GFP and CTB6^T^‐GFP are located in the cell membrane and nuclear membrane (Figure S6A, Supporting Information), suggesting that the amino acid changes do not affect the subcellular localization of CTB6. To verify whether the amino acid changes result in functional differences in CTB6's cold tolerance, we generated two types of *CTB6* CDS overexpression lines (Figure 1K,L). Compared with Towada, the seed‐setting rates of the two types of *CTB6* overexpression lines increased significantly by 20%–40% (Figure 1I,J,M,N), but no significant differences were observed between the two types of *CTB6* overexpression lines, indicating that the amino acid changes did not affect the function of CTB6. These results suggested that the promoter of *CTB6* may be the functional variant causing the differences in cold tolerance between KMXBG and Towada.

### 2.2 *CTB6* is Expressed in the Tapetum and Young Microspores and Affects Pollen Fertility Under Cold Stress

**Figure 2:**
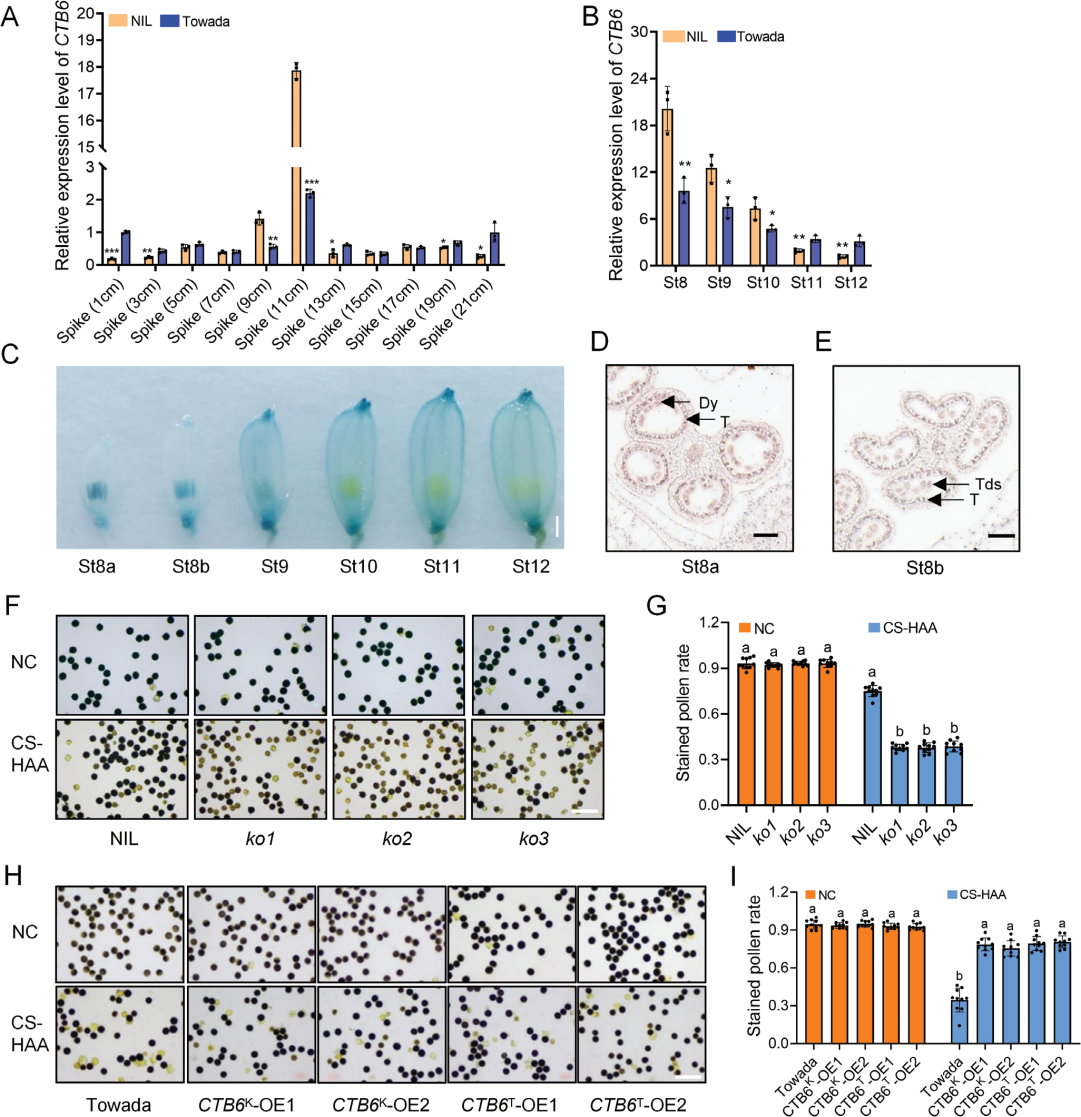
*CTB6* is expressed in the tapetum and young microspores and affects pollen fertility under cold stress. A) Relative expression of *CTB6* in panicles of different stages from NIL and Towada. B) RT‐qPCR analysis of relative *CTB6* transcript levels in different stages of anthers development. St, anther stage. In A and B, the data are means ± SD (*n* = 3), and the significant differences were determined by a two‐sided Student's *t*‐test (^*^ *P* < 0.05; ^**^ *P* < 0.01; ^***^ *P* < 0.001). *Actin1* was used as an internal reference. C) β‐Glucuronidase (GUS) staining of the *proCTB6* ^K^ *::GUS* transgenic line. Scale bar = 1 mm. D,E) mRNA in situ hybridization for *CTB6* in St8a D) and St8b E) anthers. Dy, dyad cell; Tds, tetrads; T, tapetum. Scale bar = 40 µm. F,G) The pollen fertility F) was evaluated by I_2_‐KI staining and stained pollen ratio G) of NIL and *CTB6* knockout lines under NC and CS‐HAA. Scale bar = 10 µm. H,I) The pollen fertility H) evaluated by I_2_‐KI staining and stained pollen ratio I) of Towada and *CTB6* overexpression lines under NC and CS‐HAA. NC, normal condition. Scale bar = 10 µm. In G and I, the data are means ± SD (*n* = 10), and the significance of the difference was calculated with a one‐way ANOVA analysis–Duncan test.

To clarify the tissue‐specific expression pattern of *CTB6*, we examined its expression in spikes and anthers in the NIL and Towada at different developmental stages. *CTB6* was highly expressed in 11 cm spikes and at the St8 stage of anthers (**Figure** **2**A,B). It was observed that when spikes grew to 11 cm, some anthers had already reached the St8 stage, which is the most temperature‐sensitive period for the second meiotic division of microsporocytes.^[^ ^20^ ^]^ Additionally, *CTB6* was also expressed in all the examined tissues, including the leaf, leaf sheath, and stem (Figure S6B, Supporting Information). Furthermore, using a GUS protein driven by the *CTB6* promoter, the GUS signal was highly detected in anthers at the St8a and St8b stages (Figure 2C). The result of in situ hybridization assay showed that *CTB6* was expressed in the tapetum and young microspores (Figure 2D,E). These results suggested that *CTB6* may be involved in the development of the tapetum and microspores under cold stress conditions.

Further investigation of pollen fertility in *CTB6* transgenic lines after cold stress showed that the number of fertile pollen grains in *CTB6* knockout lines was significantly lower than that in the NIL (Figure 2F,G). However, the number of fertile pollen grains in *CTB6* overexpression lines was significantly higher than that in Towada (Figure 2H,I). Additionally, no significant difference was observed in the morphology of pistils between *CTB6* knockout lines and overexpression lines after cold stress (Figure S7, Supporting Information). These results indicated that *CTB6* affects pollen fertility under cold stress conditions.

### 2.3 *CTB6* Regulates Tapetum Degradation Through Mediating ROS Homeostasis

**Figure 3:**
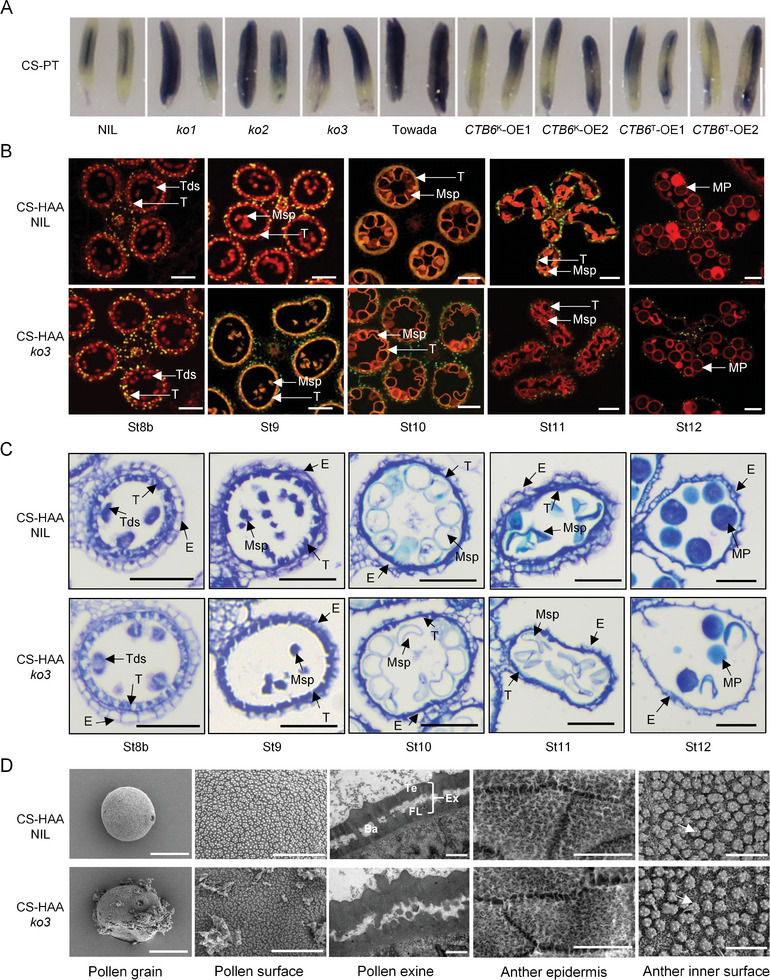
Effects of cold stress on anthers development and pollen formation in the NIL and *CTB6*‐*ko3* knockout line. A) NBT staining of the anthers from *CTB6* transgenic lines and their wild types under CS‐PT. Scale bar = 500 µm. B) Terminal deoxynucleotidyl transferase‐mediated dUTP nick end labeling (TUNEL) assay in NIL and *CTB6*‐*ko3* knockout line under CS‐HAA. Red fluorescence indicates the cell nucleus stained by PI (propidium iodide), and yellow fluorescence indicates the TUNEL‐positive signal. Scale bar = 100 µm. E, epidermis; Tds, tetrads; T, tapetum; Msp, microspore; MP, mature pollen. C) Observation of anther development in NIL and *CTB6*‐*ko3* knockout line under CS‐HAA. Scale bar = 50 µm. D) Structural observations of pollen grains and anthers in NIL and *CTB6*‐*ko3* knockout line under CS‐HAA. Pollen grain; scale bar = 20 µm, pollen surface; scale bar = 5 µm, pollen exine; scale bar = 500 nm, anther epidermis; scale bar = 20 µm, anthers inner surface; scale bar = 2 µm. Te, tectum; Ba, baculae; Ex, exine. Ubisch body (arrows) on the anther inner surface.

The tapetum provides essential nutrients for the developing pollen grain, and proper PCD of the tapetum is crucial for pollen formation.^[^ ^21^ ^]^ It was reported that LTPs play roles in maintaining ROS homeostasis.^[^ ^22^ ^]^ To investigate whether *CTB6* affects ROS accumulation in anthers, we performed nitroblue tatrazolium (NBT) staining on anthers of *CTB6* transgenic lines after cold stress. The anthers of *CTB6* knockout lines were darker in color, whereas those of *CTB6* overexpression lines were lighter (**Figure** **3**A), indicating that more ROS accumulated in the anther of *CTB6* knockout lines after cold stress, while less ROS accumulated in the anther of *CTB6* overexpression lines.

TdT‐mediated dUTP Nick‐End Labeling (TUNEL) is a method used to detect DNA breaks in apoptosis, which can specifically and accurately locate apoptotic cells.^[^ ^23^ ^]^ We conducted TUNEL analysis on the anthers of NIL and *CTB6‐ko3* knockout line. Yellow TUNEL signals were detected for the first time in the tapetum of the St8b stage in the *CTB6‐ko3* knockout line, and signal intensity was enhanced in the tapetum at the St9 stage. At stages St11 and St12, the yellow signal was undetectable. In contrast, in the NIL, strong TUNEL signal was observed at stage St10, with some slight signal present at the St11 stage (Figure 3B). These results demonstrate that the accumulation of ROS leads to the premature degradation of the tapetum in the *CTB6* knockout lines.

The premature degradation of the tapetum can adversely affect its development, leading to a reduction in its thickness.^[^ ^5^, ^18^ ^]^ Simultaneously, RT‐qPCR analysis revealed that the expression levels of genes involved in tapetum development, including *ABCG15*, *ABCG26*, and *TDR*, were significantly lower in the anther of the *CTB6‐ko3* knockout line compared with those in the NIL (Figure S8, Supporting Information). Furthermore, we conducted a slice observation of anthers at different developmental stages. Under normal conditions, no significant abnormality in anthers development were observed between the NIL and the *CTB6‐ko3* knockout line (Figure S9A, Supporting Information). However, under CS‐HAA conditions, the tapetum of the *CTB6‐ko3* knockout line was significantly thinner than that of the NIL at the St10 stage. Finally, the *CTB6‐ko3* knockout line showed abnormal microspore morphology at the St12 stage, whereas the NIL produced round, starch‐rich mature pollen grains in the anther locule (Figure 3C). Additionally, we observed the morphology of pollen grains using scanning electron microscopy. The *CTB6‐ko3* knockout line exhibited abnormal pollen grain morphology and irregular sporopollenin deposition on the pollen grain surface compared with the NIL (Figure 3D). Transmission electron microscopy observations of the internal morphology of pollen grains showed that the external wall of pollen grains in the *CTB6‐ko3* knockout line was abnormal (Figure 3D). However, the surface structure of anthers and the morphology of Ubisch bodies in the *CTB6‐ko3* knockout line did not differ from that of the NIL (Figure 3D). Taken together, these results suggested that *CTB6* regulates tapetum degradation through mediating the ROS levels, facilitating the normal morphology and structure of pollen grains under cold stress conditions.

### 2.4 CTB6 Interacts with CATs to Promote CAT Activity

**Figure 4:**
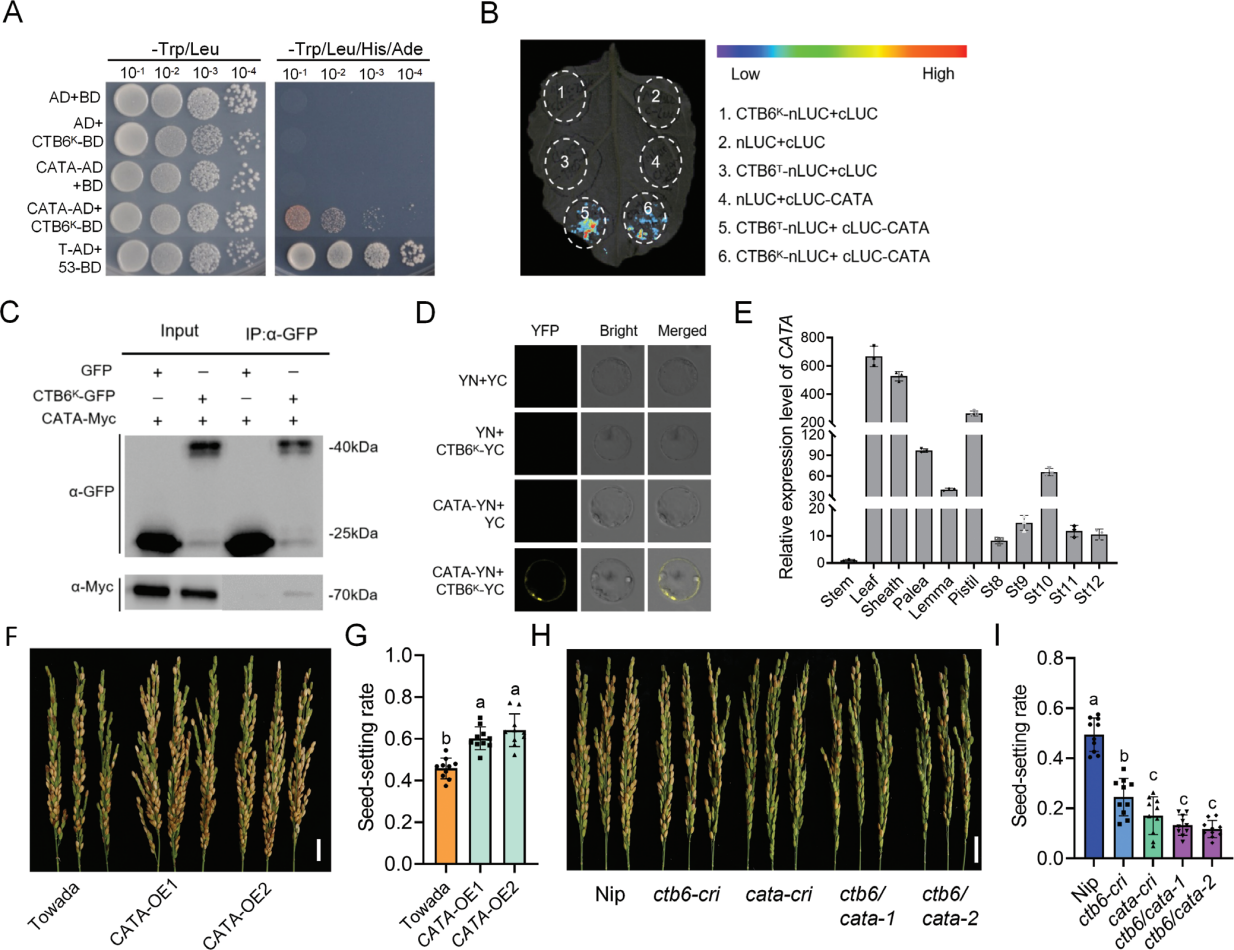
CTB6 interacts with CATA. A) Y2H assay of CTB6^K^ interacting with CATA. The vectors pGADT7 – T and pGBKT7 – 53 were used as positive controls. B) The CTB6^K^ ‐ nLUC and cLUC – CATA or CTB6^T^ ‐ nLUC and cLUC – CATA were co‐infiltrated in *N*. benthamiana leaves. C) co‐IP assay of CTB6^K^ interacting with CATA. The relative vectors were expressed in the rice protoplast. D) Constructs encoding CTB6^K^ ‐ YC and CATA – YN were co‐infiltrated in the rice protoplast. Scale bar = 10 µm. E) RT‐qPCR analysis of relative *CATA* transcript levels in various tissues of the NIL. *Actin1* was used as an internal reference. Data are means ± SD (*n* = 3). F) The phenotype of panicles of *CATA* overexpression lines grown under CS‐HAA. Scale bar = 2 cm. G) Seed‐setting rates of Towada and *CATA* overexpression lines under CS‐HAA. H) The phenotype of panicles of *ctb6*, *cata*, and *ctb6*/*cata* knockout lines under CS‐HAA. Scale bar = 2 cm. I) Seed‐setting rates of Nip, *ctb6*, *cata*, and *ctb6*/*cata* knockout lines under CS‐HAA. In G and I, the data are means ± SD (*n* = 10), and the significance of the difference was calculated with a one‐way ANOVA analysis–Duncan test.

To elucidate the molecular mechanism by which *CTB6* regulates ROS homeostasis, we conducted a yeast two‐hybrid (Y2H) assay to screen for interacting proteins and identified catalases A (CATA) (**Figure** **4**A; Table S3, Supporting Information). Rice CATs include CATA, CATB, and CATC.^[^ ^24^ ^]^ Point‐to‐point validation in yeast showed that CTB6 interacted not only with CATA but also with CATB and CATC (Figure S10A, Supporting Information). Cold‐induced expression analysis revealed that the expression level of *CATA* was higher than that of *CATB* and *CATC* under cold stress conditions (Figure S10B, Supporting Information). Therefore, we focused on CATA for subsequent studies. The luciferase (LUC) assay demonstrated that both CTB6^K^ and CTB6^T^ could interact with CATA in vivo (Figure 4B), the coimmunoprecipitation(co‐IP) assay demonstrated that CTB6^K^ interacted with CATA in vivo (Figure 4C). The bimolecular fluorescence complementation (BiFC) assay indicated that CTB6^K^ and CATA mainly interacted on the cell membrane (Figure 4D).

Expression pattern analysis of *CATA* showed that its expression level gradually increased with the development of spikes and reached the highest at the St10 stage of anther development (Figure 4E; Figure S10C, Supporting Information). Subcellular localization revealed that CATA was primarily localized in the cell membrane (Figure S10D, Supporting Information).

To validate the function of *CATA* in cold tolerance at the booting stage, we obtained two *CATA* overexpression lines (Figure S11A, Supporting Information). Cold tolerance evaluation showed that the *CATA* overexpression lines had higher seed‐setting rates than Towada under CS‐HAA, suggesting that overexpressing *CATA* could enhance cold tolerance at the booting stage (Figure 4F,G). In addition, we evaluated cold tolerance for *CATB* and *CATC* knockout lines and found that both *CATB* and *CATC* knockout lines were cold‐sensitive (Figure S12A–F, Supporting Information).

**Figure 5:**
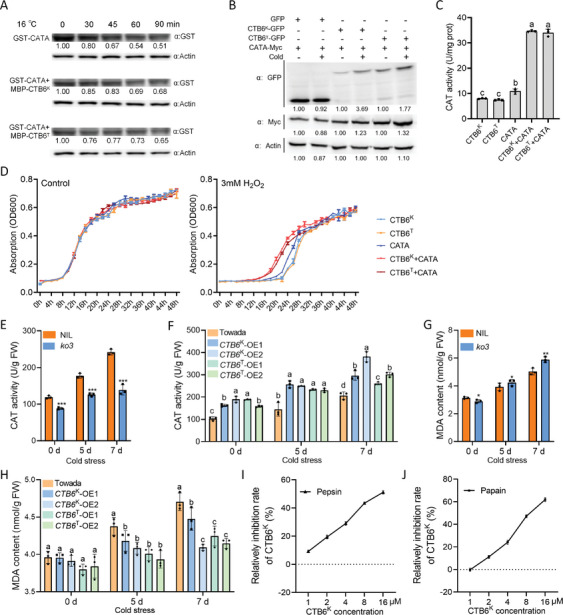
CTB6 enhances the activity of CATs. A) CATA stability assay in the absence or presence of CTB6 at low temperature (16 °C) in vitro. B) CTB6 enhances CATA stability under cold stress at 4 °C for 8 h in *N*. benthamiana leaves. C) The catalase activities of the yeast cells ectopically expressing *CTB6* ^K^/*CTB6* ^T^ and/or *CATA*. D) The growth rates of the yeast cells ectopically expressing *CTB6* ^K^/*CTB6* ^T^ and/or *CATA* under control liquid SD‐His medium or H_2_O_2_‐treated conditions. E,F) CAT activity in *CTB6*‐*ko3* knockout line E) and *CTB6* overexpression lines F) under CS‐PT. G,H) MDA contents in *CTB6*‐*ko3* knockout line G) and *CTB6* overexpression lines H) under CS‐PT. In C, D, E, F, G, and H, the data are means ± SD (*n* = 3), and the significance differences were determined by two‐sided Student's *t*‐test (^*^ *P* < 0.05; ^**^ *P* < 0.01; ^***^ *P* < 0.001). The significance of the difference was calculated with a one‐way ANOVA analysis–Duncan test. I,J) CTB6^K^ has an inhibitory effect on pepsin I) and papain(J) activity. In I, and J, the data are means ± SD (*n* = 7).

To validate the genetic interaction between *CATA* and *CTB6*, we obtained *CATA* and *CTB6* single and double knockout lines in the Nip background (Figure S11B,C, Supporting Information). We found that the seed‐setting rate of the *CATA* and *CTB6* double knockout line was significantly lower than that of the *CTB6* knockout line, but no significant difference was observed compared with the *CATA* knockout line. When compared with Nip, all knockout lines showed decreased seed‐setting rates and cold‐sensitive phenotypes (Figure 4H,I). These results suggested that CATA functions downstream of CTB6. To further explore the biological significance of the interaction between CTB6 and CATs, we performed a cell‐free degradation assay in vitro. At low temperatures, glutathione *S*‐transferase (GST)‐CATA had a longer half‐life in the presence of maltose‐binding protein (MBP)‐CTB6 compared with GST‐CATA alone (**Figure** **5**A), suggesting that the interaction between CTB6 and CATA contributes to the maintenance of CATA stability. Moreover, an in vivo assay demonstrated that the protein level of CATA was significantly higher in the presence of CTB6 at 4 °C for 8 h compared with the control (Figure 5B).

Based on these results, we performed heterologous expression validation in a yeast system and found that when CTB6 was co‐expressed with CATA, its CAT activity was significantly higher than that of any single expression (Figure 5C). Similarly, co‐expression of CTB6^K^ with CATB or CATC significantly increased CAT activity (Figure S12G, Supporting Information). Interestingly, the growth rate of yeast strains co‐expressing CTB6 and CATs was significantly higher than that of strains expressing CTB6 or CATs alone in a culture liquid SD‐His medium containing 3 mM H_2_O_2_. However, there was no significant difference in the growth rate of yeast strains in a normal liquid SD‐His medium (Figure 5D; Figure S12H, Supporting Information). Additionally, we measured CAT activity in *CTB6* transgenic lines before and after cold stress treatment, and found that after cold stress treatment, CAT activity in the panicles of *CTB6* overexpression lines was significantly increased, and the MDA content was significantly decreased; while *CTB6* knockout lines showed the opposite trend (Figure 5E–H). These results demonstrated that CTB6 interacts with CATs and increases CAT activity under cold stress, thereby maintaining ROS homeostasis.

In *Ginkgo biloba*, LTP1 has been reported to have proteinase inhibitor activity.^[^ ^25^ ^]^ To determine whether CTB6 is capable of inhibiting proteinase activity, we performed an in vitro enzyme inhibition assay. CTB6 could effectively inhibited the activities of pepsin and papain in vitro (Figure 5I,J), suggesting that CTB6 may act as a proteinase inhibitor to effectively maintain CAT stability.

### 2.5 CTB6 Possesses Lipid‐Binding Ability

**Figure 6:**
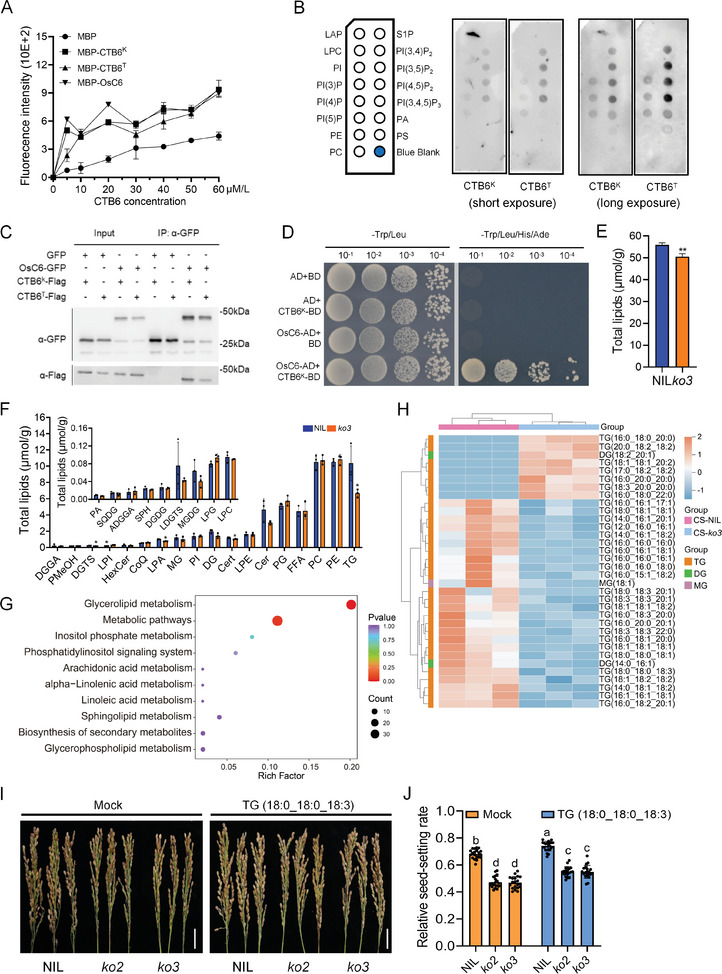
*CTB6* affects lipid content under cold stress. A) The lipid‐binding ability of recombinant CTB6 protein. The CTB6 protein was incubated with increasing amounts of 1‐pyrenedodecanoic acid. OsC6 proteins were used as positive controls, and MBP proteins were used as negative controls. B) Representative results of lipid‐protein interaction assay for MBP‐CTB6^K^ and MBP‐CTB6^T^ recombinant protein, respectively. LPA, lysophosphatidic acid; LPC, lysophosphocholine; PI, phosphatidylinositol; PI(3)P, phosphatidylinositol‐3‐phosphate; PI(4)P, phosphatidylinositol‐4‐phosphate; PI(5)P, phosphatidylinositol‐5‐phosphate; PE, phosphatidylethanolamine; PC, phosphatidylcholine; S1P, sphingosine‐1‐phosphate; PI(3,4)P_2_, phosphatidylinositol‐3,4‐bisphosphate; PI(3,5)P_2_, phosphatidylinositol‐3,5‐bisphosphate; PI(4,5)P_2_, phosphatidylinositol‐4,5‐bisphosphate; PI(3,4,5)P_3_, phosphatidylinositol‐3,4,5‐triphosphate; PA, phosphatidic acid; PS, phosphatidylserine. C) co‐IP assays of CTB6^K^ and CTB6^T^ interactions with OsC6, respectively. The relative vectors were expressed in the rice protoplast. D) Y2H assay of CTB6^K^ interacts with OsC6. E) Total lipid contents in anthers after cold stress. F) Contents of different lipid classes in anthers after cold stress. PG, phosphatidylglycerol; LPG, Lysophosphatidylglycerol; LPE, lysophosphatidylethanolamine; LPI, lysophosphatidylinositol; MG, monoacylglycerol; DG, diacylglycerol; TG, triacylglycerol; LDGTS, lysodiacylglyceryl trimethyl homoserine; DGTS, diacylglyceryl trimethylhomoserine; SQDG, sulfoquinovosyl diacylglycerol; ADGGA, acyl diacylglyceryl glucuronide; DGGA, diacylglyceryl glucuronide; DGDG, digalactosyl diacylglycerol; MGDG, mono galactosyl diacylglycerol; FFA, free fatty acid; CoQ, coenzyme Q; Cer, ceramide; Cert, phytoceramide; HexCer, glycosphingolipid; Sph, sphingosine; PMeOH, phosphatidylmethanol. G) KEGG pathway enrichment of CTB6‐related metabolic pathway. H) Heatmap shows the content levels of differential MG, DG, and TG under cold stress, which are involved in glycerolipid metabolism. Data are means ± SD (*n* = 3). Significant differences were determined by two‐sided Student's *t*‐test (^*^ *P* < 0.05; ^**^ *P* < 0.01). I) Phenotype of panicles of NIL and *CTB6* knockout lines grown with or without exogenous TG (18:0_18:0_18:3) under CS‐PT. Scale bar = 2 cm. J) Relative seed‐setting rates of the NIL and *CTB6* knockout lines with or without exogenous TG (18:0_18:0_18:3) under CS‐PT. In J, the data are means ± SD (*n* = 10), and the significance of the difference was calculated with a one‐way ANOVA analysis‐Duncan test.

Since *CTB6* encodes an LTP, we expressed CTB6 in prokaryotic cells to verify its lipid‐binding ability. According to a previous report, 1‐pyrenedodecanoic fatty acid was used as a standard, and OsC6 was used as a positive control.^[^ ^12^ ^]^ CTB6 and OsC6 had similar fluorescence absorption curves, indicating that CTB6 has lipid‐binding ability (**Figure** **6**A). Furthermore, a co‐incubation experiment of CTB6 and PIP‐Strip was carried out in vitro. CTB6 could bind phospholipid molecules, and both CTB6^K^ and CTB6^T^ exhibited the same phospholipid‐binding ability (Figure 6B). co‐IP and Y2H assays demonstrated that CTB6 could interact with OsC6 (Figure 6C,D). Considering the lipid‐binding capabilities of OsC6 and CTB6, their interaction may enhance the transport of more lipid molecules by OsC6.

### 2.6 *CTB6* Affects Lipid Content in Anthers under Cold Stress

Lipids are vital category of nutrients and synthesized in the tapetum of the anther, which are essential for anther development and the formation of mature pollen.^[^ ^26^ ^]^ To investigate the effect of *CTB6* on lipid content in anthers under cold stress conditions, we conducted lipidomic analysis of anthers from the NIL and *CTB6*‐*ko3* knockout line after cold stress. The results showed that the total lipid content in anthers of the *CTB6*‐*ko3* knockout line was significantly lower than that in the NIL after cold stress (Figure 6E; Figure S13A, Supporting Information). The decrease in triacylglycerol (TG) in the *CTB6*‐*ko3* knockout line was most pronounced compared with the NIL (Figure 6F). Kyoto Encyclopedia of Genes and Genomes (KEGG) enrichment analysis revealed that the glycerolipid metabolic pathway was a significant entry (Figure 6G). Heatmap analysis of the glycerolipid metabolic pathway showed a significant decrease in TG content in the *CTB6*‐*ko3* knockout line after cold stress (Figure 6H). Specifically, the content of TG (18:0_18:0_18:3) was significantly lower in the *CTB6*‐*ko3* knockout line than in the NIL (Figure S13B, Supporting Information). Additionally, several studies have indicated that a decrease in TG content impacts cold tolerance in rice.^[^ ^27^ ^]^ To examine whether TG is implicated in *CTB6*‐mediated cold tolerance at the booting stage, we applied exogenous TG (18:0_18:0_18:3) to the NIL and *CTB6* knockout lines under CS‐PT. The result indicated that exogenous application of TG (18:0_18:0_18:3) could increase the seed‐setting rate by 5% in the NIL, while by 10% in the *CTB6* knockout lines (Figure 6I,J). Therefore, we suggested that *CTB6* is capable of affecting cold tolerance at the booting stage by mediating the lipid content in anthers.

To investigate the reasons for the decrease in the TG content in the *CTB6*‐*ko3* knockout line, we further performed transcriptome analysis of anthers from the NIL and the *CTB6*‐*ko3* knockout line under normal and cold stress conditions. Based on the criteria of a significant difference (*p* < 0.05) and Log2Fold change > 1, a total of 1650 genes were identified as being simultaneously induced by cold stress and affected by *CTB6* (Figure S14A,B, Supporting Information). KEGG pathway enrichment analysis identified multiple prominent metabolic pathways, including lipid metabolism, carbohydrate metabolism, and the biosynthesis of other secondary metabolites (Figure S14C, Supporting Information). Combined with lipidomics data, we analyzed the genes involved in lipid metabolic pathways and found significant changes in the expression of genes associated with the glycerolipid metabolic pathway (Figure S14D, Supporting Information). RT‐qPCR analysis showed that *Os02g0802700*, *Os03g0719400*, and *Os12g0563000* had lower expression levels in the anthers of *CTB6*‐*ko3* knockout line compared with these in the NIL (Figure S14E–G, Supporting Information). These results suggested that *CTB6* may affect TG content by influencing the expression of glycerolipid‐related genes in anthers under cold stress conditions.

### 2.7 Natural Variation of *CTB6* in Rice

**Figure 7:**
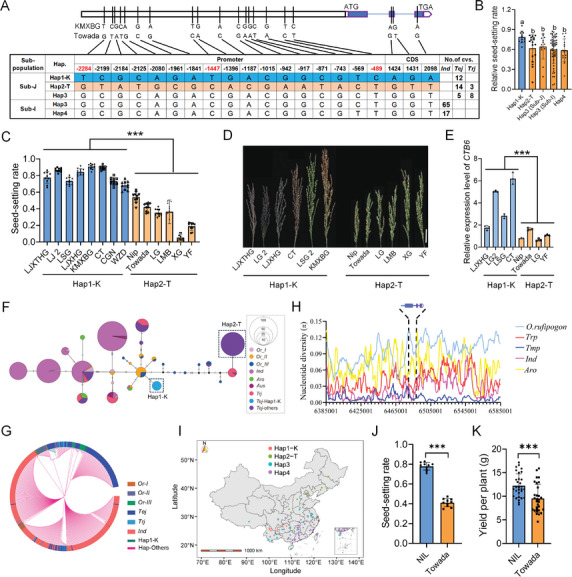
Origin and domestication of *CTB6*. A) Haplotypes of *CTB6* in 124 rice germplasms. *Tej*, temperate *japonica*; *Trj*, tropical *japonica*; *Ind*, *indica*. SNPs in the promoter and exon of *CTB6* from KMXBG and Towada. B) Relative seed‐setting rates of Hap1‐K and other haplotypes (Hap2‐T, Hap3 (Sub‐J), Hap3 (Sub‐I) and Hap4) of *CTB6* haplotypes in *japonica* under CS‐DW. C,D) Seed‐setting rates C) and panicles D) of rice germplasms carrying Hap1‐K, and Hap2‐T of *CTB6* haplotypes in *japonica* rice under CS‐HAA. Data are means ± SD (*n* = 10). LJXTHG, lijiangxintuanheigu; LG2, ligeng2; LJXHG, lijiangxiaoheigu; LSG, lengshuigu; KMXBG, kunmingxiaobaigu; CT, chongtui; CGN, cungunuo; WZD, wuzidui; LG, linguo; LMB, liming B; XG, xingguo; YF, yufu. E) Relative cold‐responsive levels of *CTB6* in rice germplasms carrying Hap1‐K and Hap2‐T of *CTB6* haplotypes under CS‐PT for 4 hours. Data are means ± SD (*n* = 3). F) Haplotype network of *CTB6*. Circle size is proportional to the number of samples for a given haplotype. G) Phylogenetic tree of *CTB6*. The color of the outer circle indicates different groups. The color of the inner branch indicates the haplotype. The green and red colors of the branches represent Hap1‐K and Hap‐Others, respectively. H) Nucleotide diversity of *CTB6* in cultivars and wild rice. (I) Distribution of *CTB6* haplotypes in China. J) Seed‐setting rate between NIL and Towada under CS‐HAA. Data are means ± SD (*n* = 10). K) Yield per plant between NIL and Towada under CS‐HAA. Data are means ± SD (*n* = 30). In B, the significance of the difference was calculated with a one‐way ANOVA analysis–Duncan test. In C, E, J, and K, the significant differences were determined by a two‐sided Student's *t*‐test (^*^ *P* < 0.05; ^**^ *P* < 0.01; ^***^ *P* < 0.001).

To explore the favorable haplotype of *CTB6*, we conducted a haplotype analysis based on the SNPs in the promoter and exon regions of *CTB6* using 124 and 971 cultivated rice accessions from the public data.^[^ ^28^ ^]^ The *japonica* subgroup contained two main haplotypes: Hap1‐K, which contained KMXBG; and Hap2‐T, which contained Towada (**Figure** **7**A; Figure S15A, Supporting Information). The relative seed‐setting rate of Hap1‐K varieties was significantly higher than that of varieties containing other haplotypes under CS‐DW conditions (Figure 7B). We selected *japonica* varieties containing Hap1‐K and Hap2‐T to further evaluate their cold tolerance under CS‐HAA conditions. The results showed that the cold tolerance of varieties containing Hap1‐K was significantly higher than that of varieties containing Hap2‐T (Figure 7C,D). Subsequently, we examined the promoter activities of Hap1‐K and Hap2‐T types of *CTB6* using GUS staining. The results indicated that the promoter activity of the Hap1‐K type was higher than that of the Hap2‐T type (Figure S15B, Supporting Information). In addition, the relative cold‐responsive levels of *CTB6* in Hap1‐K varieties were significantly higher than those in Hap2‐T varieties (Figure 7E).

Based on the above results, three SNPs (SNP‐2284, SNP‐1447, and SNP‐489) within the promoter region of *CTB6* were considered as potential natural variations associated with cold tolerance at the booting stage. Through a promoter activity assay using dual‐luciferase reporters, we found that the promoter segment containing SNP‐489 of *CTB6* ^K^ consistently exhibited higher promoter activity compared with that of *CTB6* ^T^. We further conducted site‐directed mutagenesis assay and found that the mutation in *proCTB6* ^T489C^ enhanced promoter activity compared with *proCTB6* ^T^ (Figure S15C, Supporting Information). These results suggested that SNP‐489 is a potential functional variation responsible for the differential expression levels of *CTB6*.

To investigate the origin and evolution of Hap1‐K, we constructed haplotype network and phylogenetic tree analyses of *CTB6* using 748 cultivated and 45 wild rice accessions. The haplotype network indicated that Hap1‐K originated directly from wild rice (Figure 7F), and the phylogenetic tree showed that Hap1‐K is more closely related to wild rice (Figure 7G). To determine whether *CTB6* underwent selection during rice domestication, we analyzed the nucleotide diversity of *CTB6* using publicly available data.^[^ ^28^ ^]^ Analysis of the gene region and the 100 kb segment of the flanking regions revealed that the nucleotide diversity of the *CTB6* gene region was significantly reduced in temperate *japonica* rice (Figure 7H), indicating that *CTB6* may have been selected in temperate *japonica* rice. A neutrality test showed that the nucleotide diversity of *CTB6* in temperate *japonica* rice (π = 0.0046) was significantly lower than that in tropical *japonica* rice (π = 0.02272) and wild rice (*O. rufipogon I*, π = 0.0571; *O. rufipogon II*, π = 0.06611; *O. rufipogon III*, π = 0.1124), and Tajima's *D* of *CTB6* was significantly negative in temperate *japonica* rice (Figure S15D, Supporting Information). These results indicated that *CTB6* was positively selected in temperate *japonica* rice. Furthermore, geographical distribution analysis showed that the favorable haplotype Hap1‐K was retained only in rice germplasm distributed in the Yunnan‐Guizhou Plateau region of China (Figure 7I; Figure S15E, Supporting Information). The proportion of Hap1‐K was relatively low in improved varieties (Figure S15F, Supporting Information), indicating that *CTB6* still holds considerable potential for breeding utilization.

Additionally, we evaluated the agronomic traits of the NIL and Towada under CS‐HAA conditions to assess the breeding potential of *CTB6*. The seed‐setting rate of the NIL increased by 35% compared with Towada and showed a significantly higher yield per plant under CS‐HAA conditions (Figure 7J,K). Simultaneously, plant morphological and agronomic traits of the *CTB6* transgenic lines were evaluated under CS‐HAA conditions. Statistical results showed that *CTB6* had no adverse effects on other traits except for the difference in seed‐setting rate (Figure S16A–K, Supporting Information).

## 3 Discussion

LTPs are widely present in higher plants and have been reported to participate in plant growth and development, as well as response to biotic and abiotic stress.^[^ ^29^ ^]^ However, there have been no reports on the involvement of LTPs in cold tolerance at the booting stage of rice. Here, we demonstrated that an LTP, CTB6, is involved in cold tolerance at the booting stage. *CTB6* knockout lines exhibit decreased seed‐setting rates and a cold‐sensitive phenotype under cold stress conditions (Figure 1A–D). Overexpression of *CTB6* ^K^ and *CTB6* ^T^, two alleles of *CTB6*, in the cold‐sensitive cultivar, Towada, enhances cold tolerance at the booting stage (Figure 1I–N). These results suggest that *CTB6* positively regulates cold tolerance at the booting stage in rice.

In both rice and *Arabidopsis*, ROS can act as signaling molecules to regulate the PCD of the tapetum.^[^ ^15^ ^]^ The tapetum is a unique secretory cell layer in the anther and is closely associated with the development of microspores. It provides essential nutrients, including carbohydrates and lipids, which are necessary for microspore development and the formation of the outer wall of the pollen.^[^ ^30^ ^]^ Abnormal PCD in the tapetum ultimately leads to a decrease in pollen fertility and cold tolerance.^[^ ^5^, ^21^ ^]^ However, the molecular mechanisms by which plants regulate ROS levels and the PCD of the tapetum in response to cold stress are not yet fully understood. In this study, we found that *CTB6* is highly expressed in the tapetum and young microspores of the anthers (Figure 2D,E). After cold stress, the *CTB6* knockout lines accumulated more ROS in the anthers compared with the NIL, and signals of tapetum apoptosis were detected at the St8b stage (Figure 3A,B). This led to premature degradation of the tapetum and a decrease in the expression of genes associated with tapetum development, which ultimately resulting in abnormal pollen grain morphology (Figure 3D; Figure S8, Supporting Information). Meanwhile, we verified that CTB6 interacts with CATs (Figure 4A–D; Figure S10A, Supporting Information) and demonstrated that CTB6 can promote the stability and enzymatic activity of CATs through in vitro and in vivo assays (Figure 5; Figure S12G,H, Supporting Information).

Based on the above results, we conclude that CTB6 is capable of maintaining ROS homeostasis and preventing abnormal degradation of the tapetum by enhancing the stability of CATs. While a controlled level of ROS is essential for signaling processes that facilitate normal pollen development.^[^ ^31^ ^]^ Under normal conditions, the loss‐of‐function of ABA‐activated protein kinase 2 (SAPK2) leads to a decrease in ROS levels in anthers, resulting in the delay of tapetum degradation and a reduction in the number of fertile pollen grains in rice.^[^ ^32^ ^]^ Additionally, ROS play a crucial role in pollen tube development. In *Arabidopsis*, RBOHH and RBOHJ are essential for the growth of pollen tubes. Mutations in both *RBOHH* and *RBOHJ* result in reduced ROS levels within the cells and impaired pollen tube growth.^[^ ^33^ ^]^ Therefore, the molecular mechanisms underlying the dual role of ROS in plant development should be explored in more depth.

Through sequence homology analysis, we found that both CTB6 and its homologous proteins contain eight conserved cysteine residues within the LTP domain (Figure S5B, Supporting Information). Notably, some protease inhibitors, such as EcRati, also contain these eight cysteine residues in the LTP domain. Additionally, LTP1 from *Ginkgo biloba* not only has lipid‐binding activity but also exhibits partial non‐competitive inhibitory affects on aspartic and cysteine proteinases.^[^ ^25^ ^]^ Here, we demonstrate that CTB6 has the function of inhibiting proteinase activity (Figure 5I,J). As CTB6 interacts with CATs, it is proposed that CTB6 contributes to maintaining the stability of CATs, thereby alleviating the excessive ROS in the tapetum of the anthers.

Lipids are essential nutrients for normal pollen grain development. TG, as a category of lipid molecules, plays a crucial role in abiotic stress. Our findings, combined with lipidomics and transcriptome data, suggest that CTB6 indirectly influence the expression of glycerolipid‐related genes to enhance the TG content in anthers and ensure normal pollen grains development. Meanwhile, the exogenous application of TG (18:0_18:0_18:3) could enhance the cold tolerance of *CTB6* knockout lines under cold stress conditions (Figure 6I,J). Therefore, TG is involved in *CTB6*‐mediated cold tolerance at the booting stage, highlighting its potential role in improving resilience of rice to cold stress.

LTPs serve as carriers for lipid molecules, transporting them to their desired locations.^[^ ^26^ ^]^ Several LTPs have been cloned in rice.^[^ ^9^, ^10^, ^11^, ^13^ ^]^ OsC6, as an LTP, can transfer lipid molecules from the tapetum to pollen. Loss‐function of OsC6 results in a male sterile phenotype under normal conditions. In this study, knockout of *CTB6* exhibited decreased pollen fertility only under cold stress conditions (Figure 2F,G). Given that *CTB6* is expressed in the tapetum and young microspores, possesses lipid‐binding ability, and interacts with OsC6 (Figure 2D,E and Figure 6A–D), we propose that OsC6 plays a primary role in pollen development under normal conditions. However, under cold stress conditions, OsC6 may need to carry more CTB6 to bind additional lipid molecules, thereby transporting them into pollen grains and providing the necessary safeguards for the development of pollen grains.

*CTB6* is a cold tolerance gene at the booting stage identified using a segregating population constructed from KMXBG, a cold‐tolerant variety from Yunnan province, and Towada, a cold‐sensitive variety from Japan. To investigate the functional variant region of *CTB6*, sequencing of the parents revealed that the promoter region contained 17 SNPs and the coding region contained 3 SNPs, resulting in two non‐synonymous amino acid changes (Figure 7A; Figure S5C, Supporting Information). Alterations in the *CTB6* coding region did not affect its biological function, as shown by phenotypic identification and functional validation of the lipid‐binding ability of CTB6^K^ and CTB6^T^ (Figure 1I–N and Figure 6A,B). At the same time, varieties containing Hap1‐K were more tolerant to cold and exhibited a higher degree of cold response compared with the varieties containing Hap2‐T (Figure 7C–E; Figure S15B, Supporting Information). Therefore, we believe that variation in the promoter region of *CTB6* is possibly the main reason for the differences in cold tolerance between the parents. The results of haplotype analysis and promoter activity assay indicated that the SNP‐489 in the promoter region of *CTB6* is a potential functional variation (Figure S15C, Supporting Information). The SNP‐489 was included within the AH1 motif (CAAT(A/T)ATTG), which is the binding motif of the HD‐Zip transcription factors (TFs).^[^ ^34^ ^]^ Consequently, we hypothesize that the differential binding of HD‐Zip TFs to the promoters of *CTB6* ^K^ and *CTB6* ^T^ might affect the expression of *CTB6* in rice varieties.

**Figure 8:**
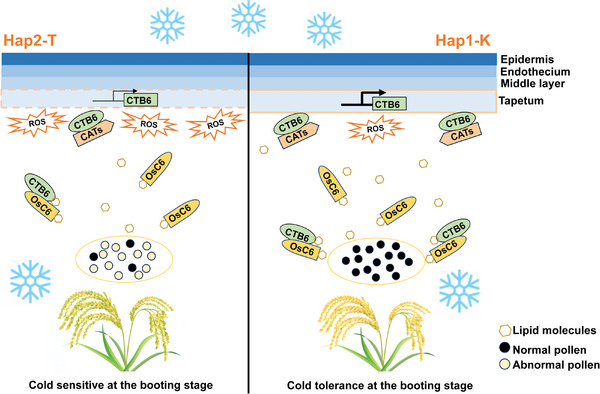
The proposed biological role of CTB6 at the booting stage under cold stress. Cold‐induced expression of *CTB6* in Hap1‐K varieties was higher than that of in Hap2‐T varieties. CTB6 interacts with CATs to regulate ROS homeostasis and prevents abnormal degradation of the tapetum. Additionally, CTB6 contains the lipid‐binding ability and affects the lipid content in anthers. CTB6 interacts with OsC6, the interaction may facilitate the transport of lipid molecules by OsC6, providing essential lipids for the normal development of pollen grains under cold stress conditions.

In this study, we have cloned a novel cold tolerance gene, *CTB6*, at the booting stage of rice. *CTB6* is mainly expressed in the tapetum and young microspores of anthers. CTB6 is capable of regulating tapetum development by maintaining the stability of CATs and eliminating ROS accumulation in anthers. Simultaneously, CTB6 can increase the lipid content in anthers and interact with OsC6 to supply sufficient lipid molecules for pollen grain development. The SNP‐489 variation in the promoter of CTB6 may increase its expression in Hap1‐K varieties under cold stress conditions (**Figure** **8**). More importantly, *CTB6* can promote the adaptation of temperate *japonica* rice to cold climates. The NIL containing *CTB6* had a higher yield per plant under cold stress conditions.

## 4 Experimental Section

### Plant Materials

The NIL was developed from the BC_7_F_5_ population by crossing the Yunnan cold‐tolerant cultivar, KMXBG (donor parent), with the Japanese cold‐sensitive cultivar, Towada (recurrent parent), through multiple backcrosses and self‐fertilization.

### Phenotype Identification

In Beijing, rice was grown under natural conditions. When the plants reached the booting stage, the panicles were treated with deep water irrigation (CS‐DW) and phytotron (CS‐PT) at a temperature of 16 °C for 7 days. After treatment, the plants were transplanted to the field to recover until the final harvest for phenotypic analysis. Finally, the relative seed‐setting rate of the rice was analyzed. In Yunnan Province, rice was grown in Kunming, where the average temperature was 16 °C. The rice was subjected to high‐altitude areas cold stress (CS‐HAA) during the entire growth period using natural low temperatures. Finally, the seed‐setting rate of the rice was studied.

For the exogenous application of TG, the NIL and *CTB6* knockout lines were grown in water with or without exogenous TG (18:0_18:0_18:3) (CAS: 88286‐50‐4, China) at a concentration of 2 mg L^−1^ under CS‐PT for 7 days.

To evaluate cold tolerance at the seedling stage, the rice seedlings were grown in a nutrient solution in a greenhouse (28 °C day/25 °C night and 65% relative humidity) for 2 weeks. Then, the seedlings were transferred to a cold chamber at 4 °C for 3–4 days. Survival rates were recorded after a 7 days recovery period at 28 °C.

### RNA Extraction and RT‐qPCR

During the vegetative and reproductive growth stages, various tissues, including stems, leaves, spikes of different lengths, and anthers at different developmental stages, were harvested. Total RNA was extracted using TRIzol™ Reagent (Invitrogen, USA) according to the manufacturer's protocol. The total RNA was then reverse‐transcribed using the TRUEscript RT kit (Aidlab, China, PC5402). RT‐qPCR analyses were performed on an ABI 7500 real‐time PCR instrument using TB Green® Premix Ex Taq™ II (Takara, Japan, RR820A) following the manufacturer's instructions. *Actin1* was used as the internal control. The primers used for the RT‐qPCR experiments in this study were listed in Table S4 (Supporting Information).

### GUS Staining

The different developmental stages of fresh anthers from the transgenic lines *proCTB6* ^Hap1‐K^ *::GUS* and *proCTB6* ^Hap2‐T^ *::GUS* were harvested and added to the GUS staining solution. The samples were incubated at 37 °C for 24 h, then decolorized with 75% alcohol, and finally photographed for observation.

### Transcriptional Activity Analysis

The different length of *CTB6* promoters from KMXBG and Towada fused to the pGreenII‐0800‐LUC vector. The relevant vectors were transiently co‐expressed in *N*. benthamiana leaves via *Agrobacterium tumefaciens* strain GV3101 (pSoup)‐mediated infiltration. Then the leaves of the same unit were lysed, and LUC and REN activities were detected using the dual‐luciferase reporter gene assay kit (YEASEN, 11402ES80).

### Subcellular Localization

The coding sequences of *CTB6* ^K^, *CTB6* ^T^, and *CATA*, excluding the stop codon, were cloned into the pSuper1300 vector. Empty pSuper1300‐GFP and *35S::CATA*‐GFP vectors were separately transformed into rice protoplasts. Additionally, *35S::CTB6* ^K^‐GFP or *35S::CTB6* ^T^‐GFP was co‐transformed into rice protoplasts with *35S::PIP2*‐mCherry (a membrane marker protein with a mCherry tag). The fluorescent signals were detected 14–16 h after transfection using a confocal microscope (Zeiss LSM880).

### In Situ Hybridization

Fresh anthers from the NIL at the S8a and S8b stages were selected. After fixation for 24 h with in situ hybridization fixative, the anthers were dehydrated, embedded, and sectioned. The cDNA sequence of *CTB6* was used as a template to generate the antisense probe. In vitro, RNA transcription and labeling were performed using the DIG RNA labeling kit. The RNA in situ hybridization protocol followed the previous report.^[^ ^35^ ^]^

### Sequence and Phylogenetic Analysis

Orthologs of CTB6 were retrieved from the NCBI database (https://www.ncbi.nlm.nih.gov/), and the amino acid sequences of the putative orthologs were used for phylogenetic analysis. A phylogenetic tree was constructed using MEGA software with the neighbor‐joining method and 1000 bootstrap replications.^[^ ^12^ ^]^

### Y2H Assay

The coding sequence of *CTB6* ^K^ was cloned into the pGBKT7 (BD) vector. The coding sequences of *CATA, CATB, CATC*, and *OsC6* were cloned into the pGADT7 (AD) vector, respectively. The *CTB6* ^K^ ‐ BD construct was co‐transformed into the yeast strain AH109 with *CATA –* AD, *CATB –* AD, *CATC –* AD, or *OsC6 –* AD. The pGADT7 – T and pGBKT7 – 53 vectors were used as positive controls. The yeast clones were grown on SD‐Trp‐Leu medium at 30 °C for 3 days. The clones were then transferred to SD‐Trp‐Leu‐His‐Ade medium at 30 °C to detect interactions.

### BiFC Assay

The coding sequences of *CTB6* ^K^ and *CATA* were cloned into the cYFP (YC) and nYFP (YN) vectors, respectively. The *CTB6* ^K^ ‐ YC construct was co‐transformed into rice protoplasts with *CATA –* YN. The *CTB6* ^K^ ‐ YC and an empty YN vector, as well as *CATA –* YN and an empty YC vector, were used as negative controls. The YFP fluorescent signal was detected 14–16 h after transfection using a confocal microscope (Zeiss LSM880).

### Split‐LUC Complementation Assay

The coding sequences of *CTB6* ^K^ and *CTB6* ^T^ were cloned into the nLUC (N‐terminal nLUC) vector. The coding sequence of *CATA* was cloned into the cLUC (C‐terminal cLUC) vector. The *CTB6* ^K^ ‐ nLUC or *CTB6* ^T^ ‐ nLUC constructs with an empty cLUC vector, and *CATA –* cLUC with an empty cLUC vector were used as negative controls. The relevant vectors were transiently co‐expressed in *N. benthamiana* leaves via *Agrobacterium tumefaciens* strain EHA105‐mediated infiltration. After 3 days, 1 mM D‐luciferin (NanoLight, USA, 300‐250) was sprayed onto detached leaves and kept in the dark for 5 min. The LUC signal was detected using the Fusion FX7 instrument (Vilber, France).

### co‐IP Assay

To perform the co‐IP assay, *CTB6* ^K^ ‐ GFP, and CATA – Myc were transiently co‐expressed in rice protoplasts. Additionally, OsC6‐GFP was co‐transformed into rice protoplasts with *CTB6* ^K^ ‐ Flag or *CTB6* ^T^ ‐ Flag. Total protein was extracted using protein extraction buffer (50 mM Tris‐HCl, pH 7.5, 150 mM NaCl, 10 mM MgCl_2_, 2% [v/v] NP‐40, 1 mM DTT, 1×Cocktail). Protein samples were then incubated with 20 µL GFP‐Nanoab‐Agarose beads (Lablead, China, GNA‐20‐400) at 4 °C for 2 h with gentle agitation. Immunoblotting was performed using anti‐GFP (TransGen, HT801, 1:5000 dilution), anti‐MYC (CWBIO, CW0299M, 1:5000 dilution), anti‐Flag (Sigma, F1084, 1:5000 dilution), and anti‐Actin (ABclonal, AC009, 1:5000 dilution) antibodies, along with anti‐mouse secondary antibody (CWBIO, CW0102S, 1:5000 dilution).

### Recombinant Protein Production and Purification

The coding sequence of CATA was cloned into the pMAL‐c2G vector (adding an MBP tag), and the coding sequences of *CTB6* ^K^, *CTB6* ^T^, and *OsC6* were cloned into the pGEX‐4T‐1 vector (adding a GST tag). The relevant vectors were transformed into *Escherichia coli* strain BL21(DE3). When the OD600 reached 0.6, 0.5 mM IPTG was added, and the cultures were grown at 16 °C for 16 h. The recombinant proteins were purified according to the manufacturer's instructions.

### TUNEL Assay

Transverse sections of various developmental anther samples subjected to cold treatment were prepared for the TUNEL assay. The TUNEL assay was performed using a TUNEL kit (CF488 TUNEL Cell Apoptosis Detection Kit, Servicebio; G1504) according to the manufacturer's instructions. The samples were analyzed using a confocal laser‐scanning microscope (Eclipse C1, Nikon).

### Fluorescent Fatty Acid Binding Assay

The lipid‐binding activity of MBP‐CTB6^K^ and MBP‐CTB6^T^ was assessed according to a previous report.^[^ ^12^ ^]^ Fluorescence intensity was measured using an enzyme‐labeling instrument (Tecan Spark, Austria). The excitation wavelength was set at 343 nm, and the emission spectrum was recorded at 378 nm.

### Protein‐Lipid Overlay Assay

The protein‐lipid overlay assay with PIP strips (Echelon Biosciences, USA, P‐6001) according to the manufacturer's instructions was performed. The PIP strips were incubated with blocking buffer (TBST: 10 mM Tris‐HCl pH 8.0, 140 mM NaCl, 0.1% [v/v] Tween‐20) containing 3% BSA and gently agitated for 1 h at room temperature. The recombinant proteins, MBP – CTB6^K^ and MBP – CTB6^T^, were added and incubated overnight at 4 °C. The lipid membranes were then washed three times with gentle agitation for 10 min each. The PIP strips were incubated with anti‐MBP antibody (TransGen, HT701, 1:5000 dilution) at room temperature for 2 h with gentle shaking. Next, the PIP strips were developed using anti‐mouse secondary antibodies after 1 h at room temperature.

### Microscopy

Pollen fertility analysis and pistil morphology observations were performed 1 day before flowering under normal and cold stress conditions. The morphology of the pistil was directly observed under a microscope (C‐DSS230, Nikon). The anthers were immersed in 50% FAA solution for 1 day, then fixed anthers and the pollen grains were released onto a microscope slide, stained with 1% (w/v) I_2_‐KI solution, and examined under a light microscope (Olympus CX23). For paraffin sectioning, the anthers were fixed in the FAA solution. After dehydration through a graded ethanol series, the samples were embedded in paraffin and cut into 60–80 µm thick sections using a microtome (Leica UC7). Sections were stained with 0.5% (w/v) toluidine blue, rinsed with distilled water, dried overnight, and examined with an optical microscope (Olympus CX23). TEM (HT7800, Hitachi) and SEM (SU8100, Hitachi) were performed according to the manufacturer's instructions.

### Lipidome and Transcriptome Analyses

For lipidome analysis of the NIL and the *CTB6‐ko3* knockout line at the booting stage, total lipids were isolated from anthers subjected to CS‐PT for 7 days. Plant lipid contents were detected by MetWare using the AB Sciex QTRAP 6500 LC‐MS/MS platform. KEGG enrichment analysis was performed using Metware Cloud, a free online platform for data analysis (https://cloud.metware.cn).

For transcriptome analysis of the NIL and the *CTB6‐ko3* knockout line at the booting stage, total RNA was isolated from anthers under NC and CS‐PT conditions for 7 days. KEGG enrichment analyses of differentially expressed genes were performed using Metware Cloud.

### The Growth Rate and CAT Activity in Yeast

The pESC‐His vector was used to express Myc‐tagged CATs and Flag‐tagged CTB6^K^/CTB6^T^ in yeast strain AH109 under the control of the GAL1 and GAL10 promoters, respectively. To determine the affect of CTB6^K^ and CTB6^T^ on CAT activity in yeast, the yeast lines expressing Myc‐CATs and/or Flag‐CTB6^K^/Flag‐CTB6^T^ were harvested. To measure the growth rate, the yeast lines expressing the pESC‐His vectors were incubated in liquid SD‐His medium at 30 °C until the OD600 reached 0.1. H_2_O_2_ was then added to the liquid SD‐His medium to a final concentration of 3 mM. The yeast lines were further incubated in the liquid SD‐His medium, with and without H_2_O_2_, and the OD600 values were recorded hourly. Curves based on OD600 values were plotted to reflect growth rates. To measure CAT activity, the yeast lines were grown to the same concentration. Vector construction and CAT activity assays were performed as described previously.^[^ ^36^ ^]^

### Characterization of Proteinase Inhibitory Activities

The proteinase inhibitory activity was measured using the double‐antibody one‐step sandwich method‐enzyme‐linked immunosorbent assay (ELISA) kits (pepsin, MM‐1258W2; papain, MM‐926711O2). The detection method was conducted according to the manufacturer's instructions.

### Phylogenetic and Selection Analyses

The haplotype network and phylogenetic tree analyses of CTB6 were conducted using the data reported by.^[^ ^37^ ^]^ For the construction of the phylogenetic tree, aligned sequences were generated with MEGA7 using the neighbor‐joining method.^[^ ^38^ ^]^ The genome sequences of *CTB6*, including the 2.5 kb promoter and the coding region, were collected for evolutionary analysis. For nucleotide diversity analysis, genotype information was processed and imported into DNASP 5.10 software.^[^ ^7^ ^]^ The nucleotide diversity (Pi) and Tajima's *D* statistics were calculated using MEGA7.0 software for each rice subpopulation.

### Statistical Analysis

Statistical tests were indicated mean ± SD. And the significant differences were determined by a two‐sided Student's *t*‐test or one‐way ANOVA analysis–Duncan test. *P* < 0.05 was considered as significant. The band intensity of immunoblotting was measured with ImageJ.

## Conflict of Interest

The authors declare no conflict of interest.

## Author Contributions

G.S.L., L.J., Z.Y.W. contributed equally to this work. L.J.J., L.Z.C. designed the research. G.S.L., L.J.J. wrote the manuscript. G.S.L., L.J., Z.Y.W. performed the experiments. L.H.H., G.Z.H., G.H.F., Z.M., G.Y.S., S.R.B., Y.W., Z.A.D., S.X.M., Z.Z.Y., Z.H.L.,G.Y.M., M.W.D., Y.P.R. provided technical assistance.

## Supporting information



Supporting Information

## Data Availability

The data that support the findings of this study are available from the corresponding author upon reasonable request.
