# Supplementary material for: CTB6 Confers Cold Tolerance at the Booting Stage by Maintaining Tapetum Development in Rice
Source: Adv Sci (Weinh). 2025 Jan 22;12(10):2411357. doi: 10.1002/advs.202411357 (PMC11905004; doi:10.1002/advs.202411357)
Supplement: Supplementary file 1 — Supporting Information [file ADVS-12-2411357-s001.docx]

Supporting Information

***CTB6* Confers Cold Tolerance at the Booting Stage by Maintaining Tapetum Development in Rice**

*Shilei Gao, Jin Li, Yawen Zeng, Huahui Li, Zhenhua Guo, Haifeng Guo, Meng Zhang, Yunsong Gu, Runbin Su, Wei Ye, Andong Zou, Xingming Sun, Zhanying Zhang, Hongliang Zhang, Yongmei Guo, Wendong Ma, Pingrong Yuan, Zichao Li*^*^*and Jinjie Li*^*^


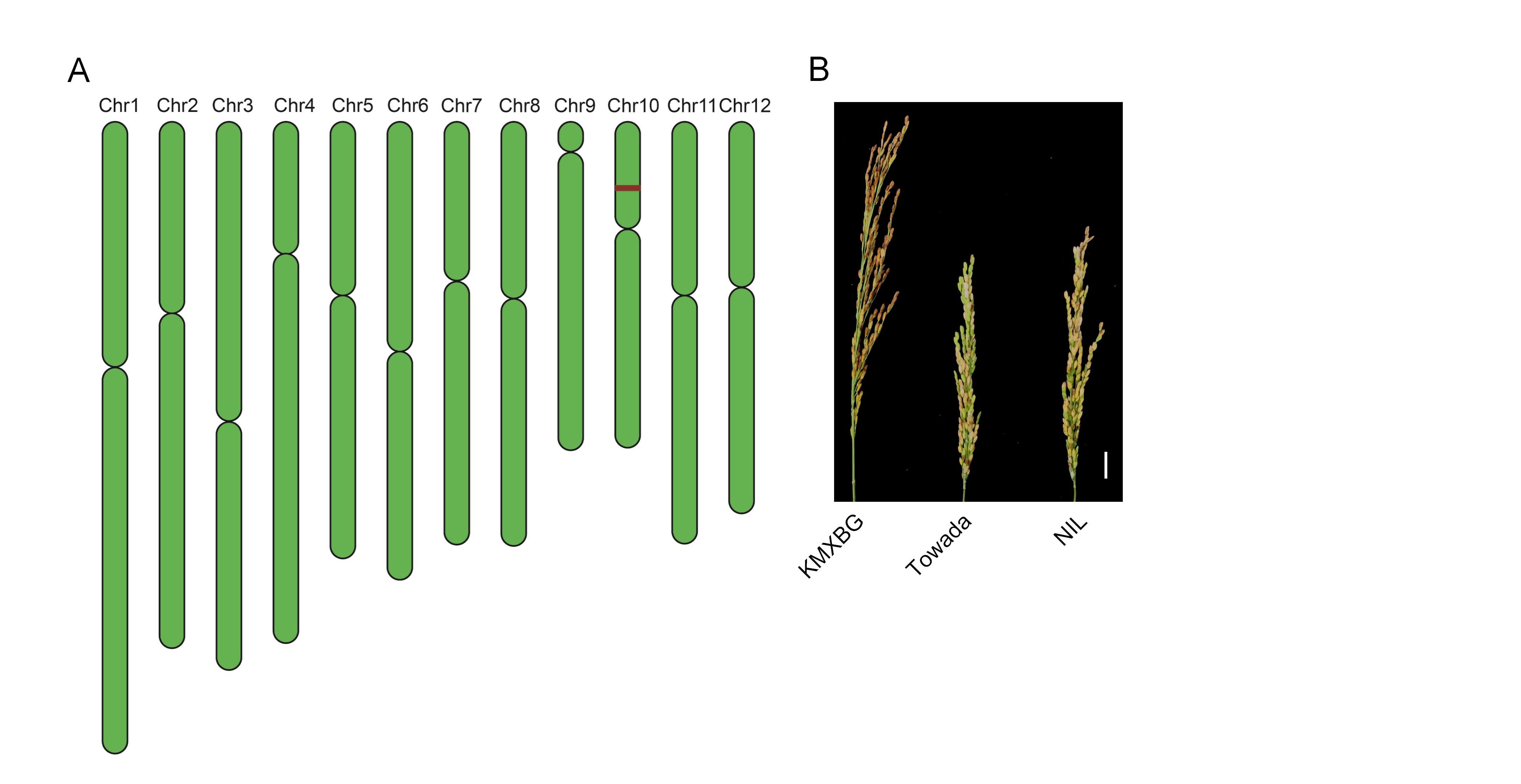


**Figure S1.** The background information regarding the NIL. A) Graphic genotype of NIL. Red region, genomic region of KMXBG; Green region, genomic region of Towada. B) Phenotype of panicles of KMXBG, Towada and NIL grown under CS-HAA. Scale bar = 2 cm.

**
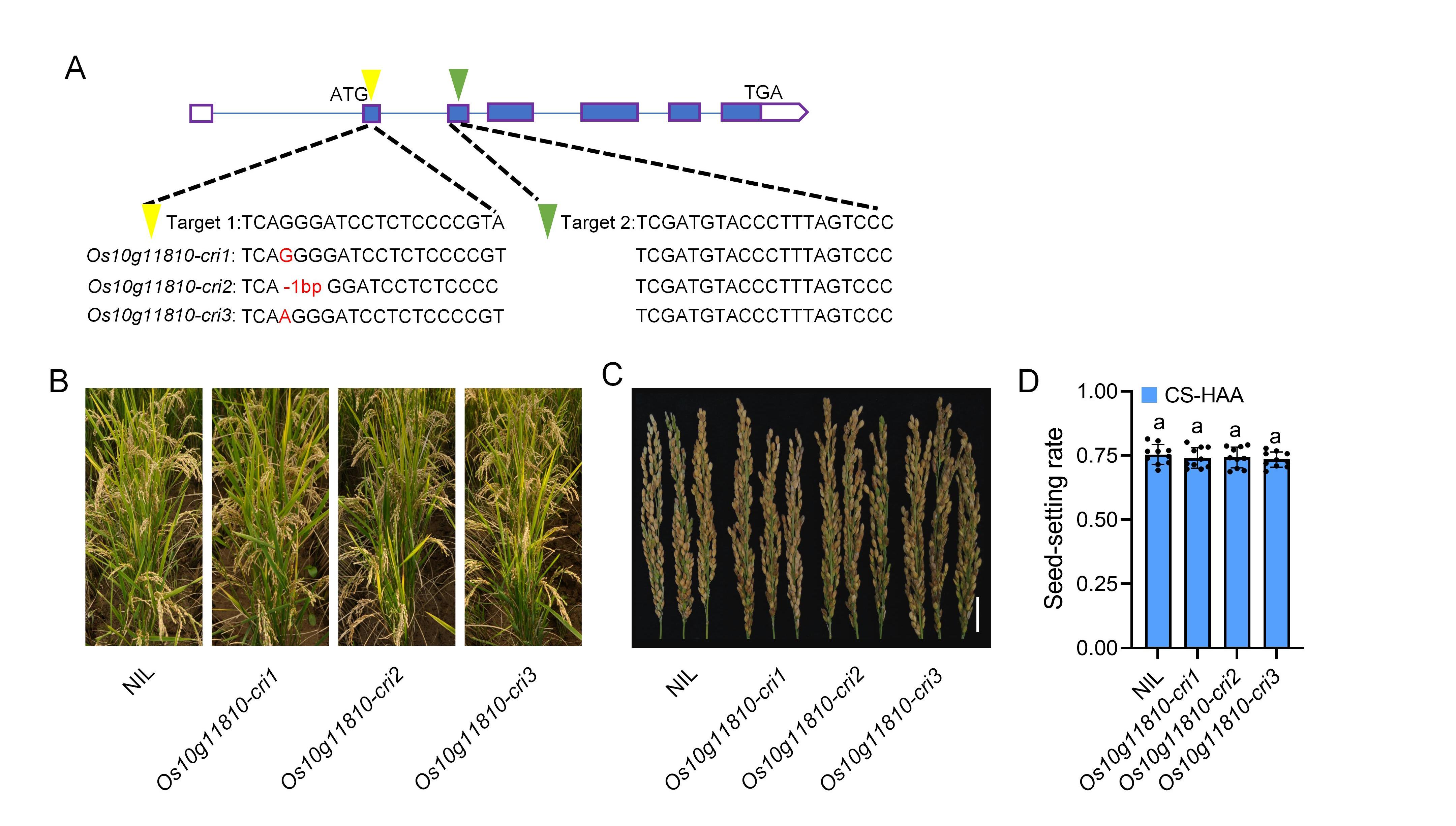
**

**Figure S2.** Identification of *Os10g11810* in cold tolerance at the booting stage. A) Gene structure and editing information for *Os10g11810*. White boxes represent the untranslated region; Intervening lines represent introns; and blue boxes represent the exon. B,C) Phenotype of plants B) and panicles C) of NIL and *Os10g11810* knockout lines grown under CS-HAA. Scale bar = 2 cm. D) Seed-setting rate of NIL and *Os10g11810* knockout lines under CS-HAA. Data are means ± SD (*n* = 10), and the significance of the difference was calculated with a one-way ANOVA analysis–Duncan test. CS-HAA, cold stress in high-altitude areas.


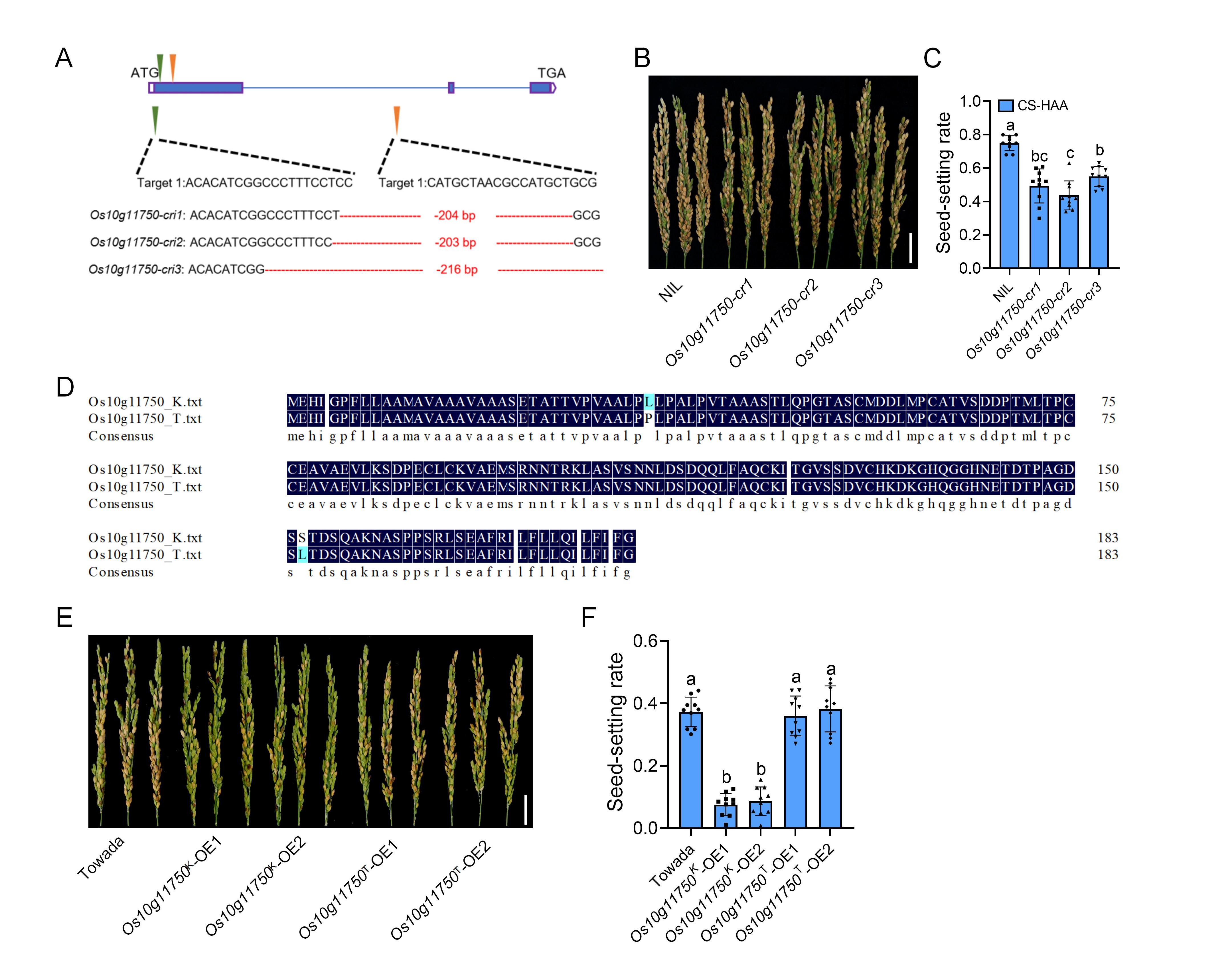


**Figure S3.** Identification of *Os10g11750* in cold tolerance at the booting stage. A) Gene structure and editing information for *Os10g11750*. White boxes represent the untranslated region; Intervening lines represent introns; and blue boxes represent the exon. B) Phenotype of panicles of NIL and *Os10g11750* knockout lines grown under CS-HAA. Scale bar = 2 cm. C) Seed-setting rate of NIL and *Os10g11750* knockout lines under CS-HAA. D) The amino acid variations of Os10g11750 in KMXBG (K) and Towada (T). E) Phenotype of panicles of Towada and *Os10g11750* overexpression lines under CS-HAA. Scale bar = 2 cm. F) Seed-setting rate of NIL and *Os10g11750* overexpression lines under CS-HAA. In C and F, the data are means ± SD (*n* = 10), and the significance of the difference was calculated with a one-way ANOVA analysis–Duncan test.


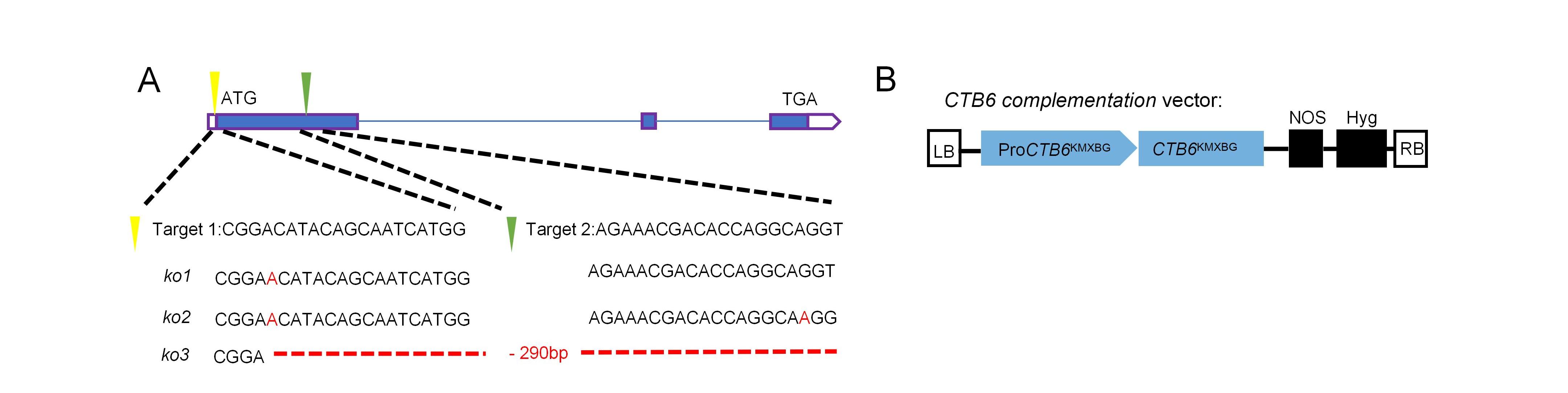


**Figure S4.** *CTB6* gene structure and the information of complementation vector. A) Gene structure and editing information for *CTB6*. White boxes represent the untranslated region; Intervening lines represent introns; and blue boxes represent the exons. B) Schematic of complementation vectors for transgenic analysis.


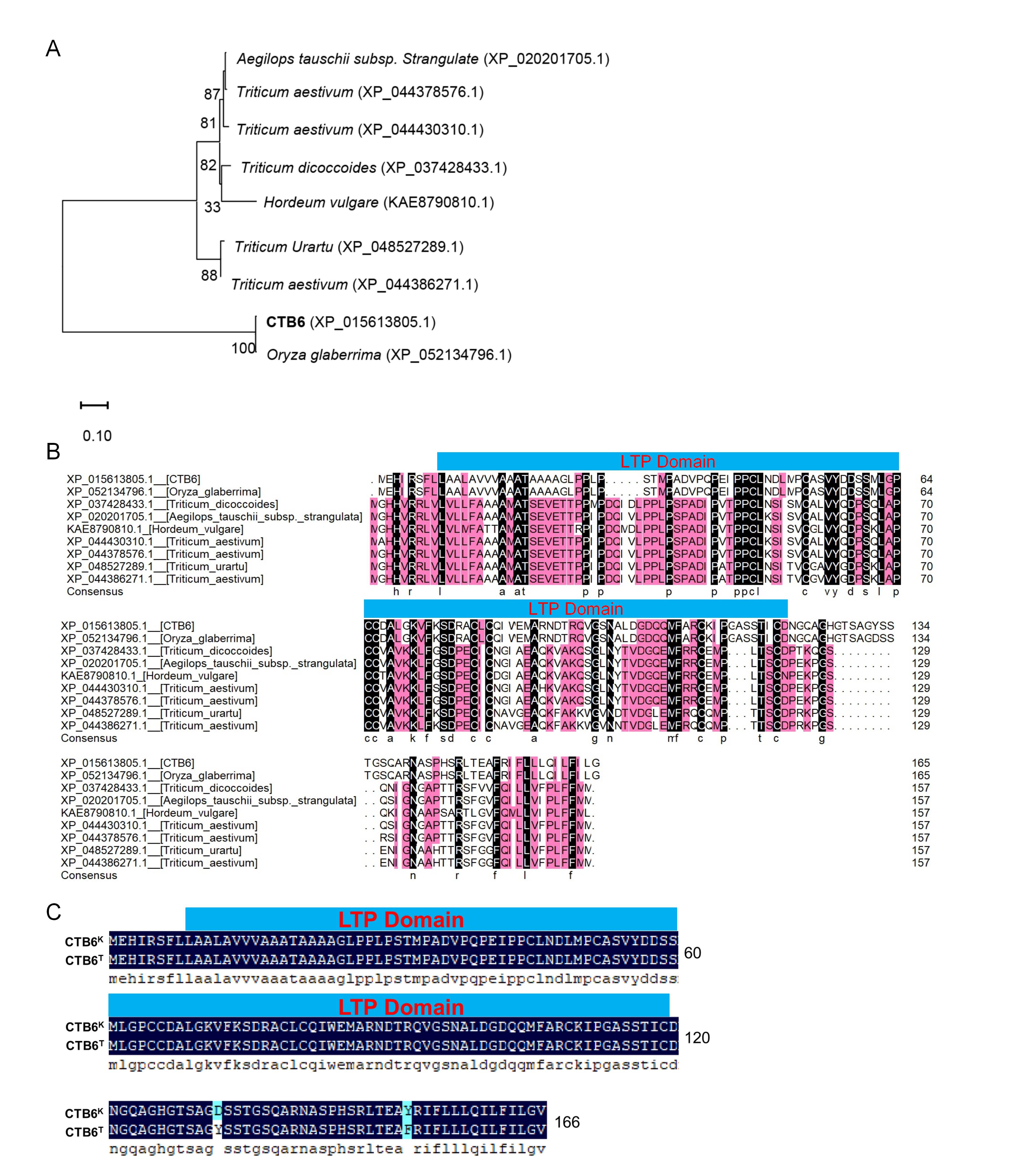


**Figure S5.** Homology analysis of CTB6**.** A) Phylogenetic tree of CTB6 and its orthologs from other species. The numbers at the nodes indicate the bootstrap support values. The scale bar represents the number of expected changes per amino acid residue. B) Amino acid sequence alignment of CTB6 homologous proteins. Amino acids with more than 75% conservation are shown in pink, and 100% conservation is shown in black. C) The amino acid variations of CTB6 (LOC_Os10g11730) in KMXBG (K) and Towada (T).

**
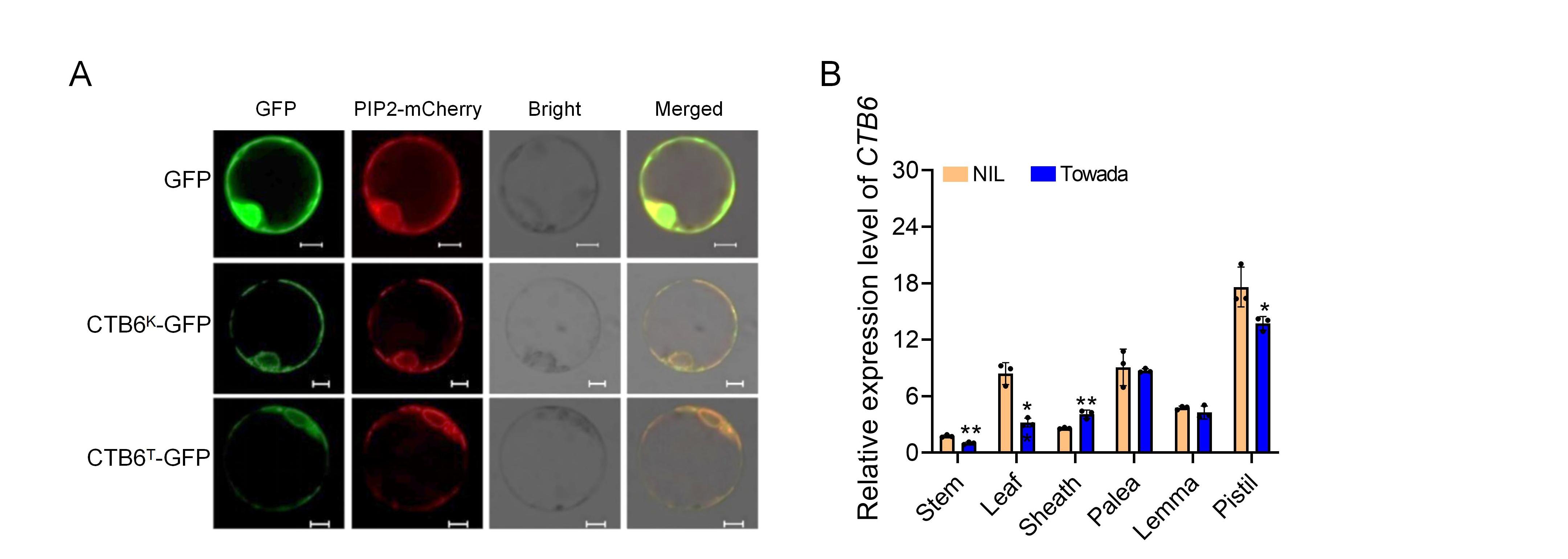
**

**Figure S6.** Expression pattern of *CTB6*. A) Subcellular localization of CTB6^K^ and CTB6^T^ by co-localization with PIP2-mCherry in the rice protoplast. Scale bar = 10 μm. B) RT-qPCR analysis of relative *CTB6* transcript levels in various tissues. *Actin1* was used as an internal reference. Data are means ± SD (*n* = 3), and the significant differences were determined by two-sided Student’s *t*-test (*, *P* < 0.05; **, *P* < 0.01).

**
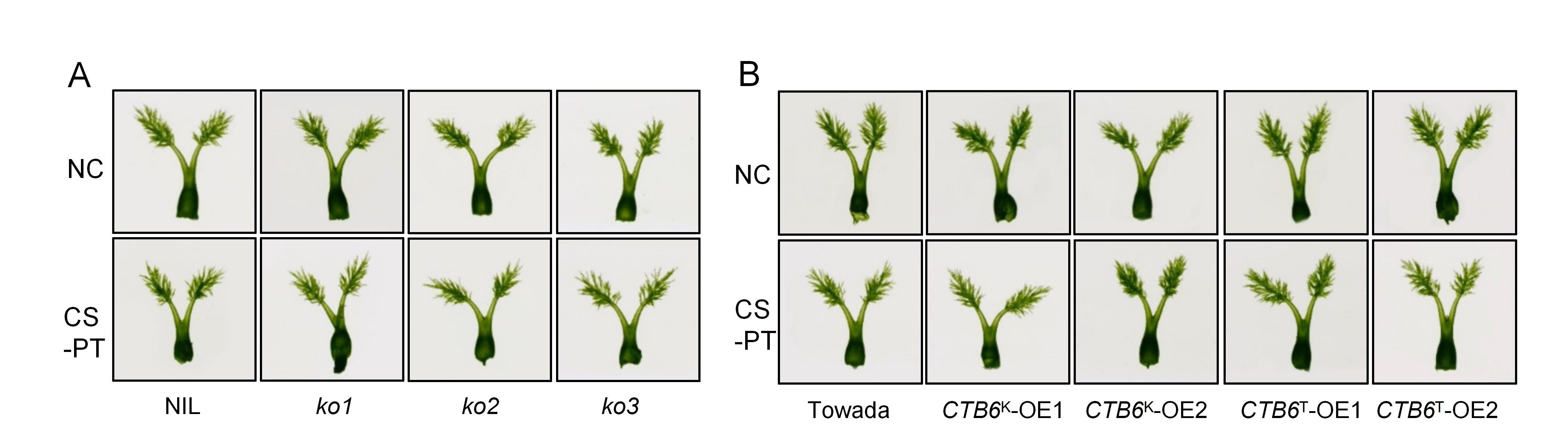
**

**Figure S7.** The phenotype of pistils of *CTB6* transgenic lines and their wild types. A) The pistil morphology of NIL and *CTB6* knockout lines under NC and CS-PT. B) The pistil morphology of Towada and *CTB6* overexpression lines under NC and CS-PT.


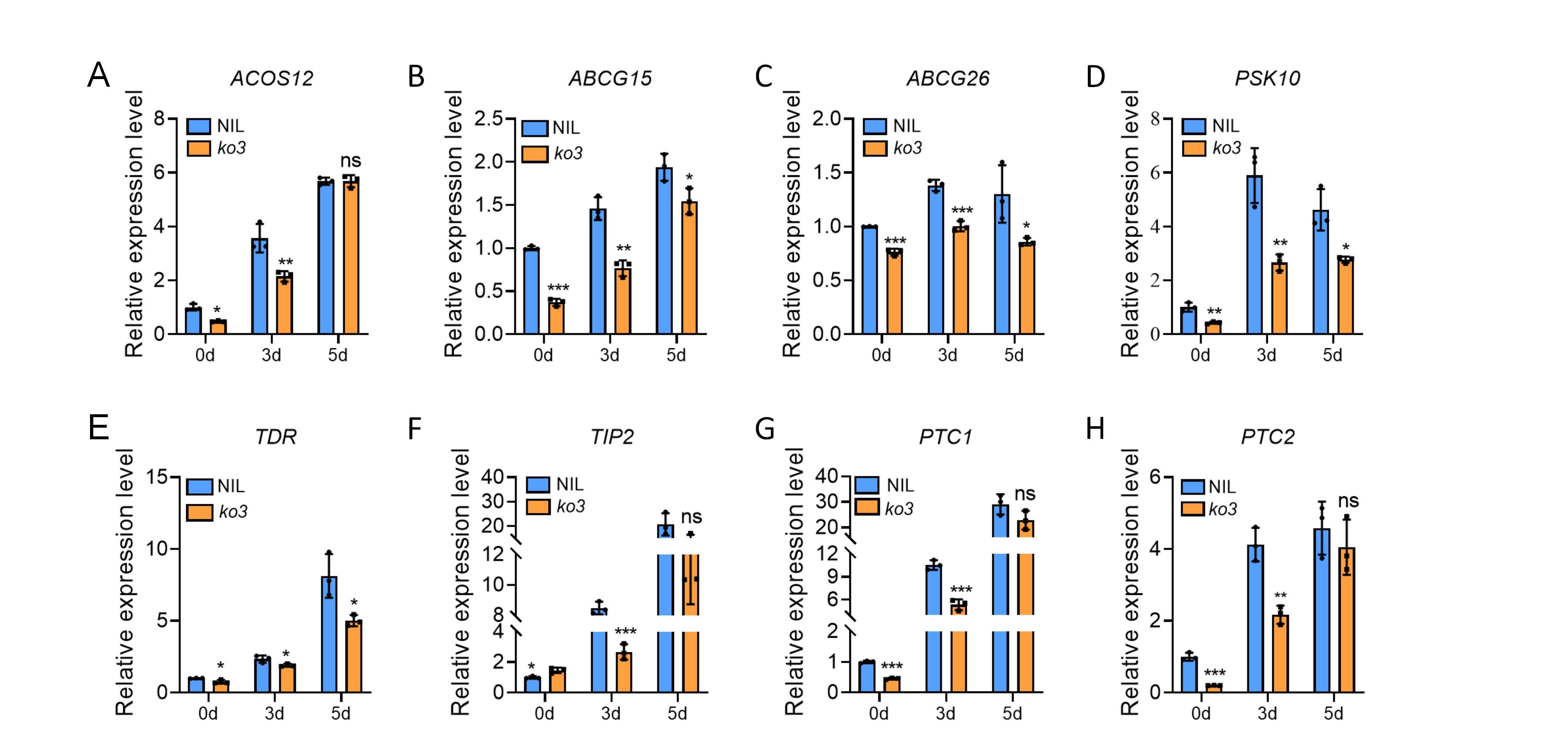


**Figure S8.** The relative expression levels of genes associated with tapetum development in the anthers of NIL and *CTB6*-*ko3* knockout line under cold stress. A-H) Lower expression levels of genes were detected in the anthers of *CTB6*-*ko3* knockout line compared with NIL. Data are means ± SD (*n* = 3), and the signiﬁcant differences were determined by a two-sided Student’s *t*-test (*, *P* < 0.05; **, *P* < 0.01; ***, *P* < 0.001).

**
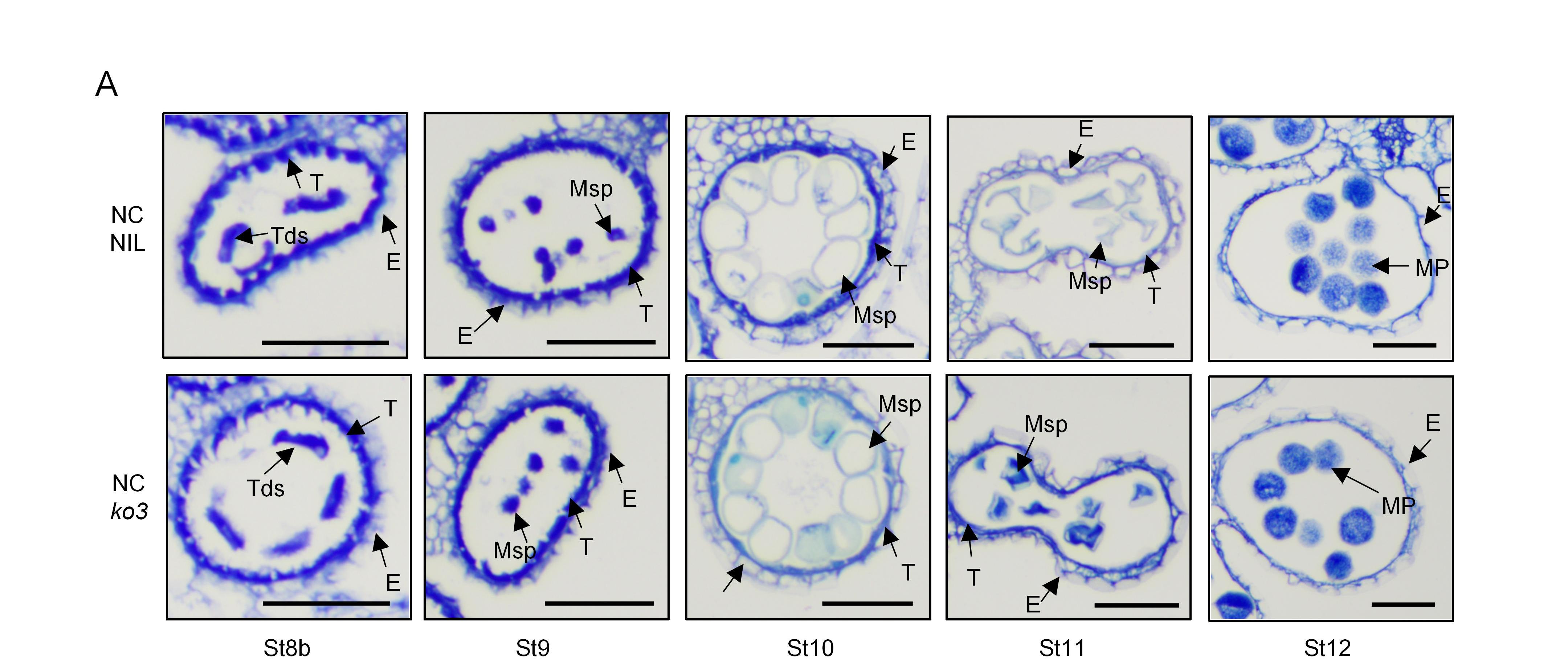
**

**Figure S9.** Histological analysis of anther development in the NIL and *CTB6-ko3* knockout line. A) Observation of anther development in the NIL and *CTB6-ko3* knockout line under NC. Scale bar = 50 μm. E, epidermis; Tds, tetrads; T, tapetum; Msp, microspore; MP, mature pollen.

**
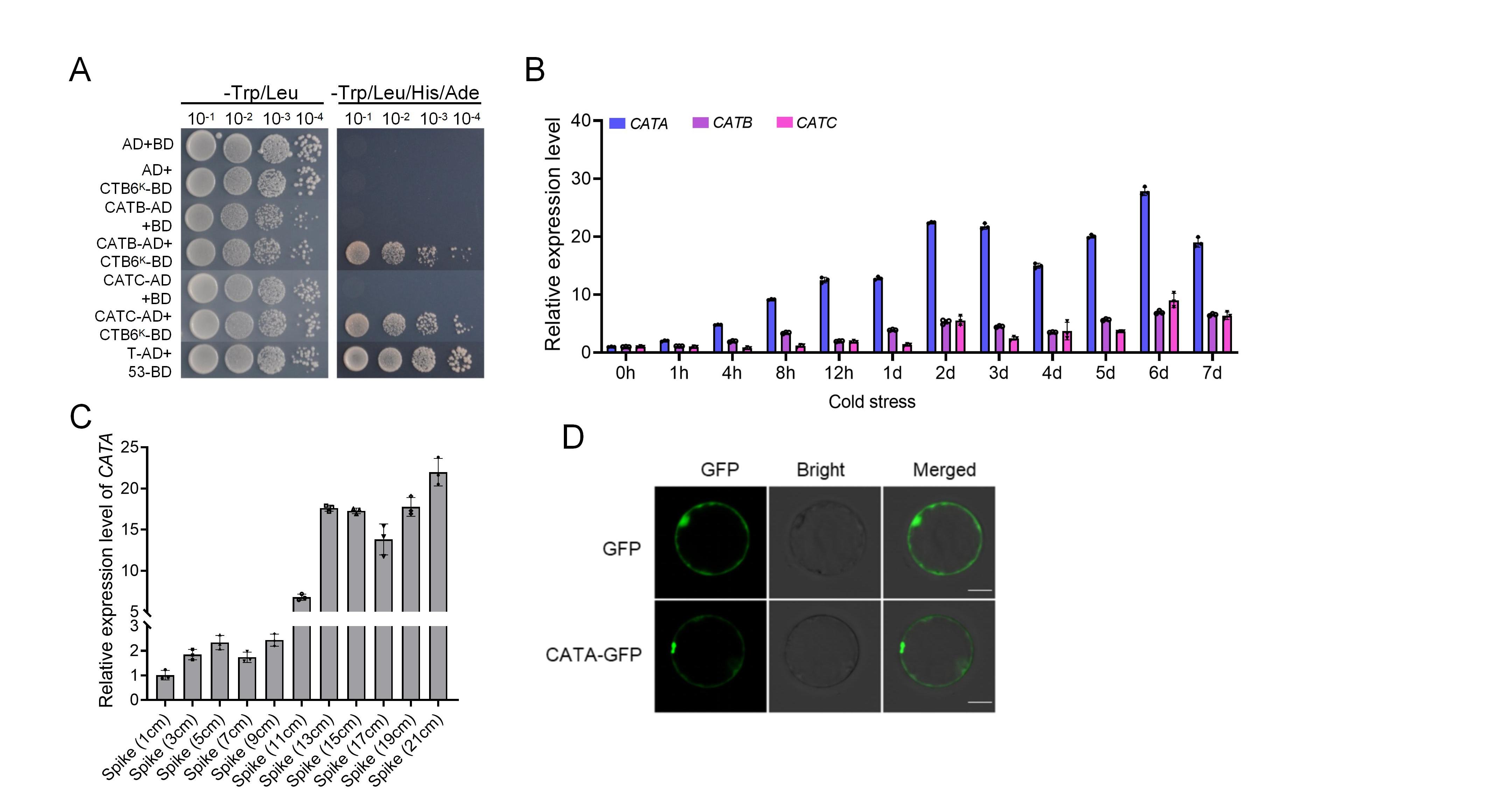
**

**Figure S10.** CTB6 interacts with CATs. A) Y2H assays of CTB6^K^ interacting with CATB and CATC, respectively. The vectors pGADT7 - T and pGBKT7 - 53 were used as positive controls. B) The *CAT's* relative expression level in panicles of NIL at different times under CS-PT. C) Relative expression level of *CATA* during the panicle development of NIL. *Actin1* was used as an internal reference. Data are means ± SD (*n* = 3). D) Subcellular localization of CATA in the rice protoplast. Scale bar = 10 μm.


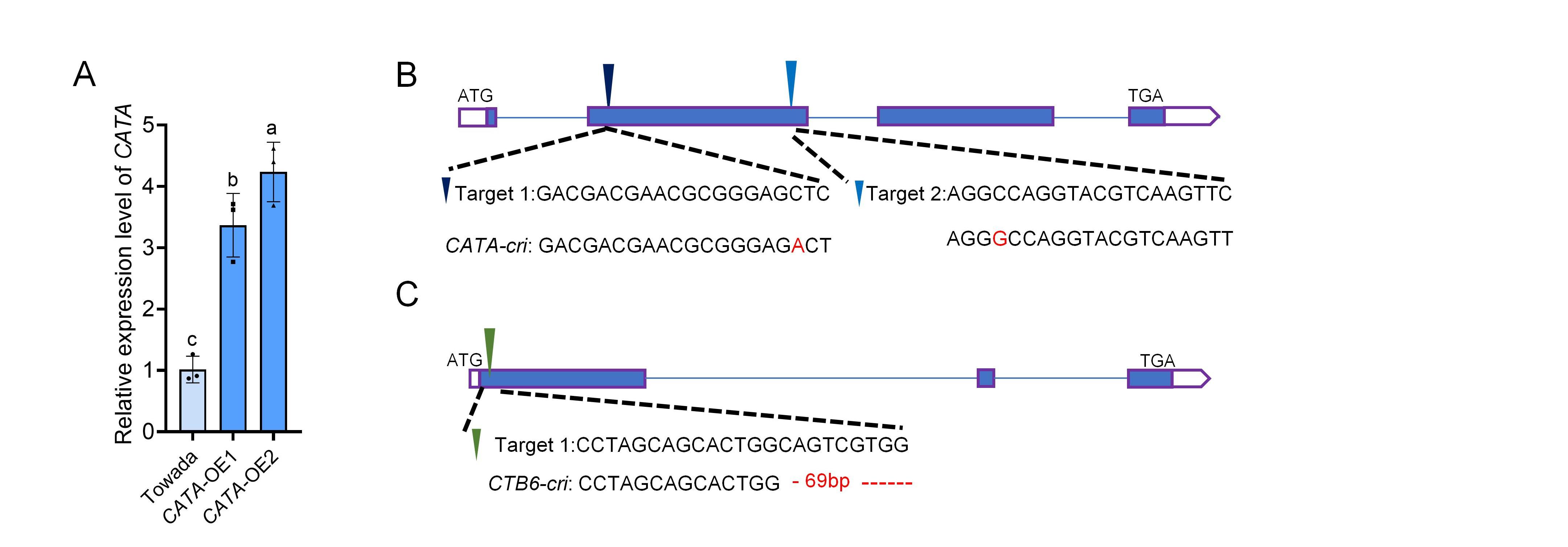


**Figure S11.** Overexpressing efficiency of *CATA* and editing information of *CATA* and *CTB6*. A) Overexpressing efficiency of *CATA* in transgenic lines by RT-qPCR. *Actin1* was used as an internal reference. B,C) Gene structure and editing information for *CATA* B) and *CTB6* C) in the Nip background. White boxes represent the untranslated region; Intervening lines represent introns; and blue boxes represent the exon. Data are means ± SD (*n* = 3), and the significance of the difference was calculated with a one-way ANOVA analysis–Duncan test.

**
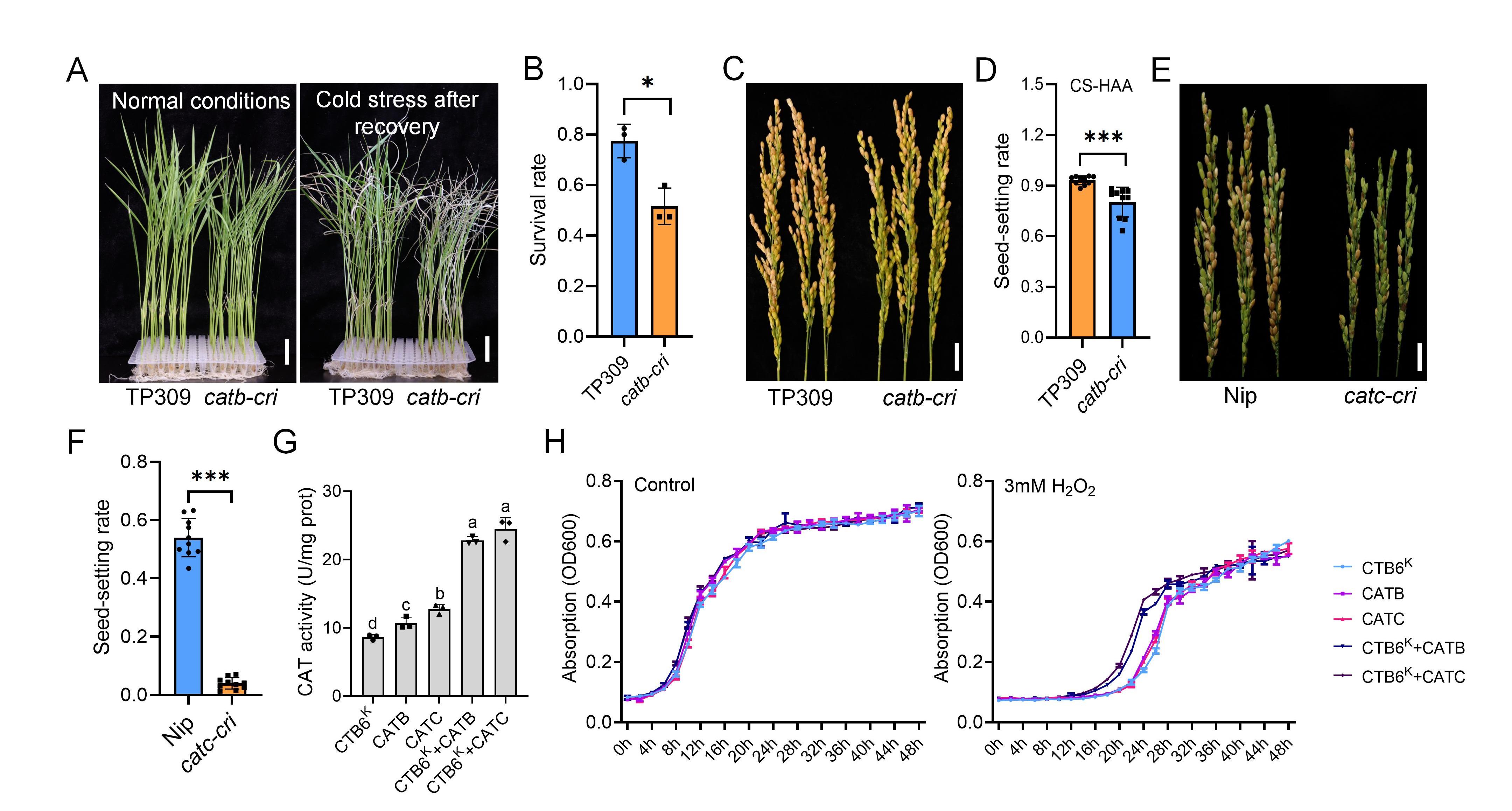
**

**Figure S12.** CATs enhance cold tolerance and reduce H_2_O_2_ accumulation. A)The seedling phenotypes of *CATB* knockout line and its wild type, TP309, under normal conditions and after cold stress. Scale bar = 2 cm. B) The survival rates of *CATB* knockout line and its wild type after cold stress. Data are means ± SD (*n* = 3), and the significant differences were determined by two-sided Student’s *t*-test (*, *P* < 0.05). C) Phenotype of panicles of TP309 and *CATB* knockout line under CS-HAA. Scale bar = 2 cm. D) Seed-setting rates of TP309 and *CATB* knockout line under CS-HAA. E) Phenotype of panicles of Nip and *CATC* knockout line under CS-HAA. Scale bar = 2 cm. F) Seed-setting rates of Nip and *CATC* knockout line under CS-HAA. In D and F, the data are means ± SD (*n* = 10), and the significant differences were determined by two-sided Student’s *t*-test (***, *P* < 0.001). G) The catalase activities of the yeast cells ectopically expressing *CTB6*^K^ and/or *CATB*/*CATC*. H) The growth rates of the yeast cells ectopically expressing *CTB6*^K^ and/or *CATB*/*CATC* under the control liquid SD-His medium or H_2_O_2_-treated conditions. In G, the data are means ± SD (*n* = 3), and the significance of the difference was calculated with a one-way ANOVA analysis–Duncan test.


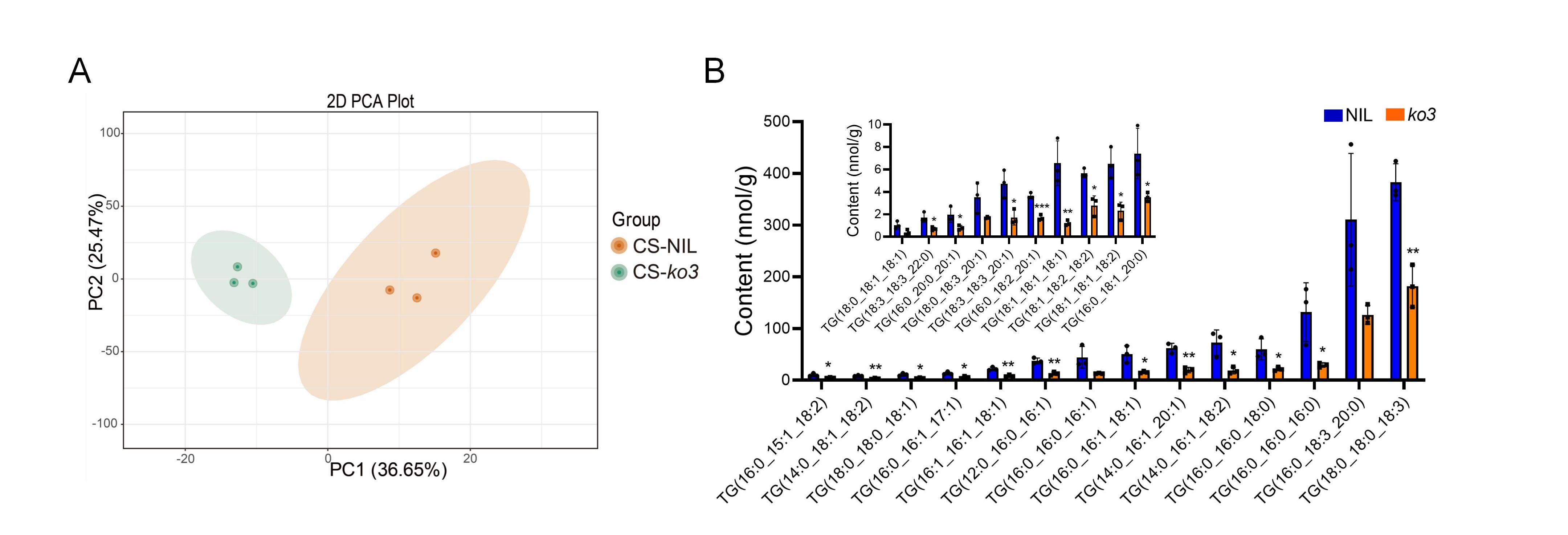


**Figure S13.** Comprehensive analysis of lipidomic profiles in anthers. A) Principal component analysis (PCA) of whole lipid compounds in anthers under cold stress. B) Contents of different triacylglycerol classes in anthers of the NIL and *CTB6-ko3* knockout lines under cold stress. The data are means ± SD (*n* = 3), and the significant differences were determined by two-sided Student’s t-test (*, *P* < 0.05; **, *P* < 0.01; ***, *P* < 0.001).


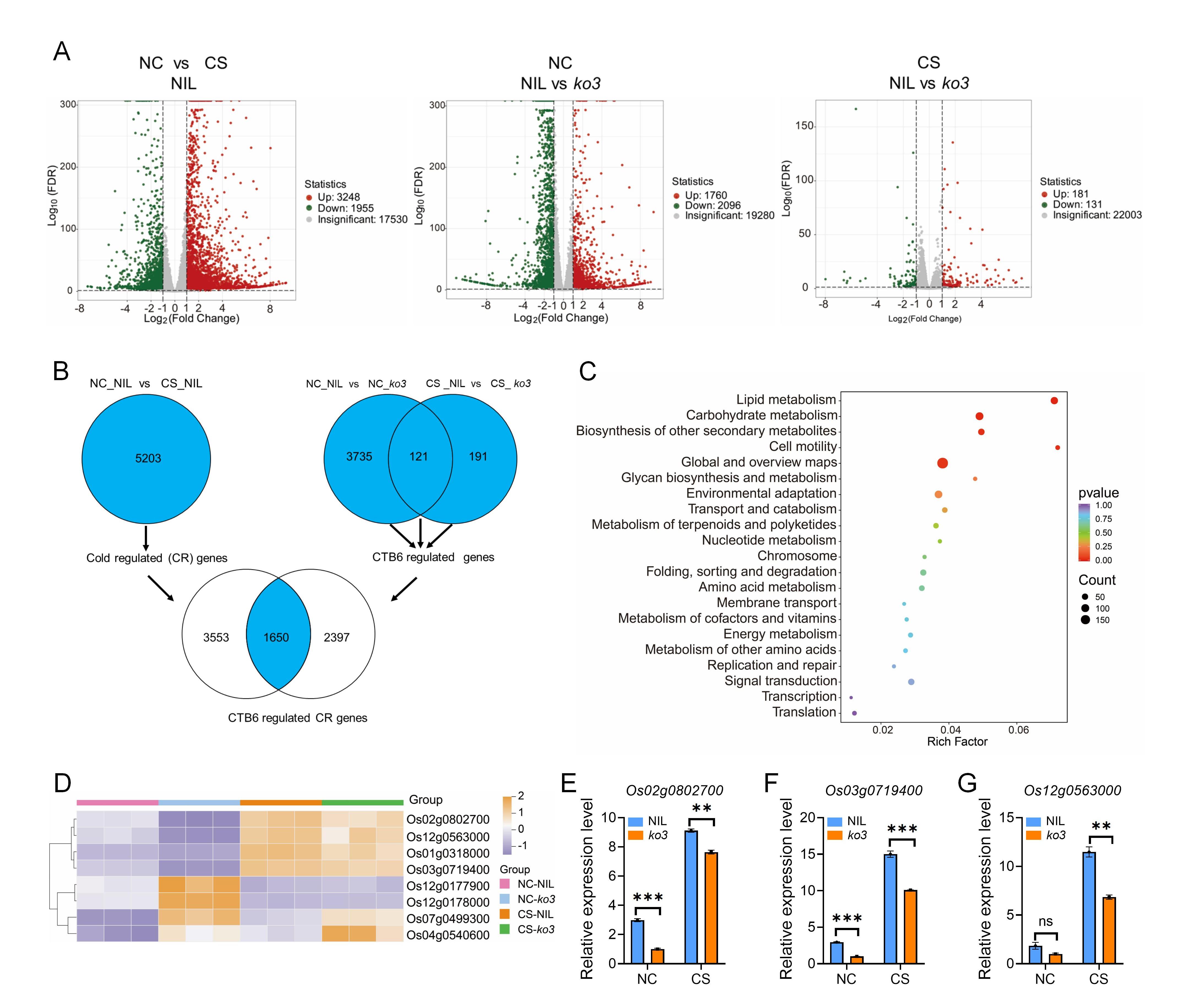


**Figure S14.** Transcriptome analyses between NIL and *CTB6-ko3* knockout line under NC and CS. A) Volcano plots representing the fold-change of DEGs in the comparison groups of CS_NIL versus NC_NIL, NC_*ko3* versus NC_NIL, and CS_*ko3* versus CS_NIL, (absolute log2Fold change > 1, adjusted *P* value < 0.05). Gray dots represent genes without significant changes in expression. Three independent experiments were performed for each sample at each time point. B) Venn diagrams representing DEGs regulated by cold (top, left), CTB6 (top, right), and both cold and CTB6 (bottom). C) KEGG pathway enrichment of the CTB6-related cold-responsive genes. D) Heatmap showing the expression levels of differentially expressed glycerolipid metabolism genes with and without cold stress, which are involved in lipid metabolism. E-G) Relative expression levels of *Os02g0802700* E), *Os03g0719400* F), and *Os12g0563000* G) in anthers of NIL and *CTB6*-*ko3* knockout line under NC and CS. Actin1 was used as an internal reference. In E, F, and G, the data are means ± SD (*n* = 3), and the signiﬁcant differences were determined by a two-sided Student’s *t*-test (*, *P* < 0.05; **, *P* < 0.01; ***, *P* < 0.001).

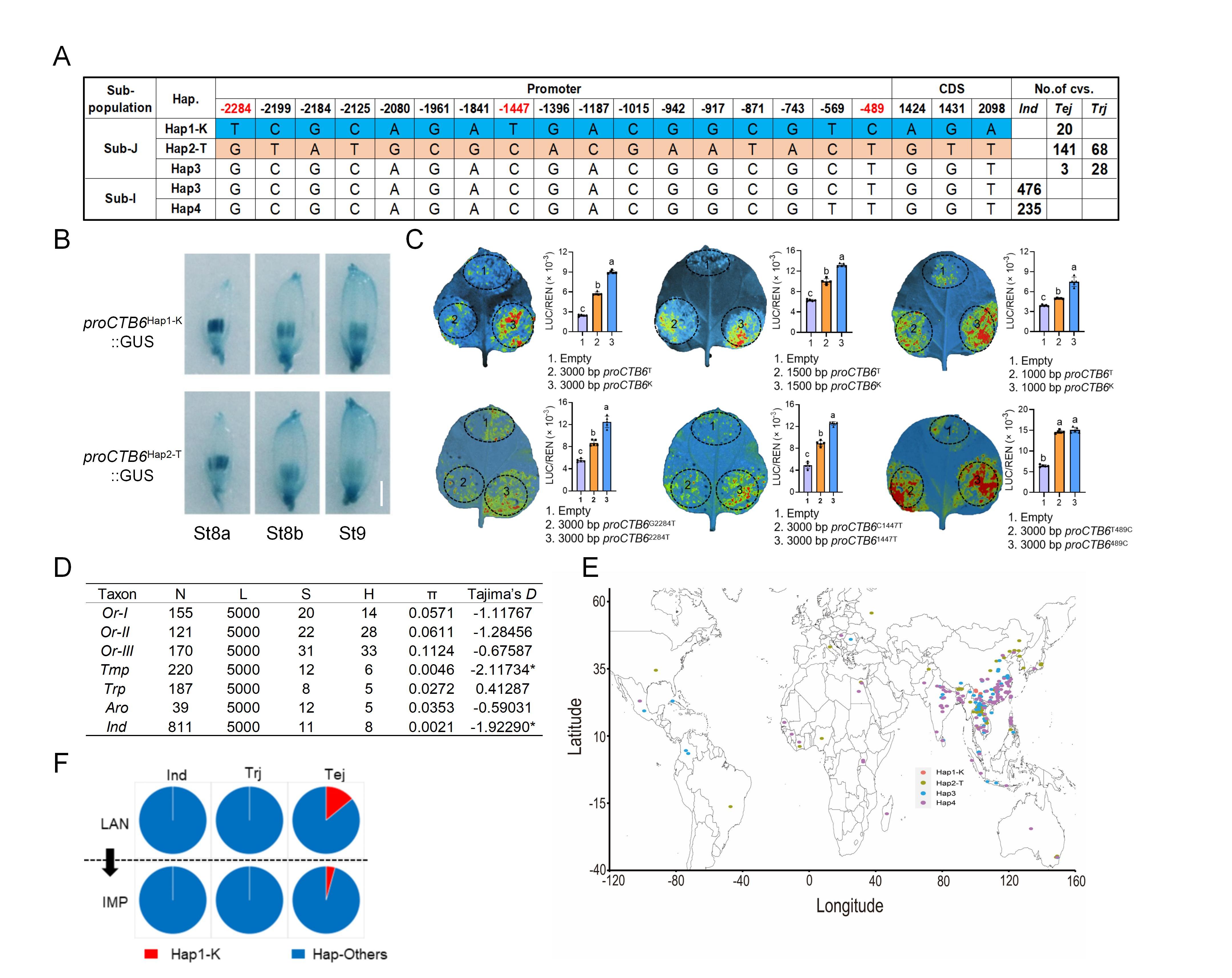


**Figure S15.** Selection analysis and allelic changes of *CTB6*. A) Haplotypes of *CTB6* in 971 rice germplasms. B) GUS staining of the anthers of *proCTB6*^Hap1-K^::GUS and *proCTB6*^Hap2-T^::GUS transgenic lines. Scale bar = 1 mm. C) Site-directed mutagenesis assay of three SNPs within the promoter region of *CTB6* using dual-luciferase reporters in *N*. benthamiana leaves. Data are means ± SD (*n* = 5), and the significance of the difference was calculated with a one-way ANOVA analysis-Duncan test. D) Estimated parameters of nucleotide diversity and Tajima’s *D* of *CTB6* and its flanking regions. E) Distribution of *CTB6* haplotypes worldwide. F) Allelic changes in *CTB6* during rice breeding. LAN, landraces varieties; IMP, improved varieties.


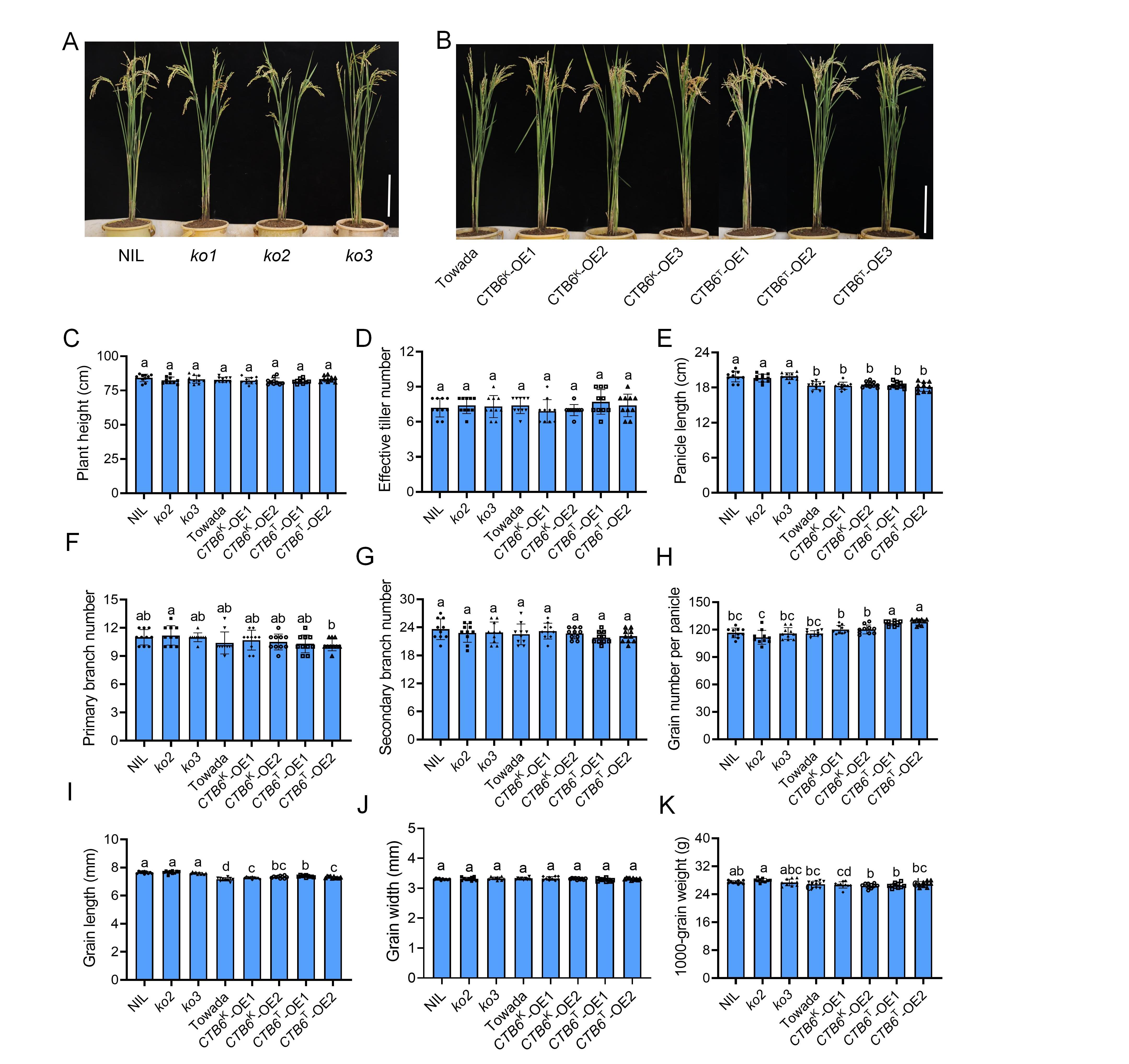


**Figure S16.** Agronomic traits of *CTB6* transgenic lines and their wild types under CS-HAA. A) Morphology of NIL and *CTB6* knockout lines under CS-HAA. Scale bar = 20 cm. B) Morphology of Towada and *CTB6* overexpression lines under CS-HAA. Scale bar = 20 cm. C-K) The plant height C), effective tiller number D), panicle length E), primary branch number F), secondary branch number G), grain number per panicle H), grain length I), grain width J), and 1,000-grain weight K) of *CTB6* transgenic lines and their wild types under CS-HAA. Data are means ± SD (*n* = 10). The significance of the difference was calculated with a one-way ANOVA analysis–Duncan test.**Table S1.** The number of SNPs in candidate genes

| ORF | The mumber of SNPs in the 2.5kb promoter region | The mumber of SNPs in the coding region |
| --- | --- | --- |
| LOC_Os10g11730 | 17 | 3 |
| LOC_Os10g11750 | 13 | 2 |
| LOC_Os10g11770 | 7 | 2 |
| LOC_Os10g11810 | 7 | 0 |

**Table S2.** Phylogenetic analysis

| Orthologs | Amino acids sequence |
| --- | --- |
| Oryza sativa Japonica Group (XP_015613805.1) | MEHIRSFLLAALAVVVAAATAAAAGLPPLPSTMPADVPQPEIPPCLNDLMPCASVYDDSSMLGPCCDALGKVFKSDRACLCQIWEMARNDTRQVGSNALDGDQQMFARCKIPGASSTICDNGQAGHGTSAGYSSTGSQARNASPHSRLTEAFRIFLLLQILFILGV |
| Oryza glaberrima (XP_052134796.1) | MEHIRSFLLAALAVVVAAATAAAAGLPPLPSTMPADVPQPEIPPCLNDLMPCASVYDDSSMLGPCCDALGKVFKSDRACLCQIWEMARNDTRQVGSNALDGDQQMFARCKIPGASSTICDNGQAGHGTSAGDSSTGSQARNASPHSRLTEAFRIFLLLQILFILGV |
| Triticum dicoccoides (XP_037428433.1) | MGHHVRRLVLVLLFAAAAMATSEVETTPPMPDQIDLPPLPSPADIPVTPPCLNSISMCALVYQDPSQLAPCCVAVKKLFGSDPECICNGIAEAQKVAKQSGLNYTVDGQEMFRRCEMPLTSCDPTKQGSQNIGNGAPTTRSFVVFQILLVFPLFFMM |
| Aegilops tauschii subsp. Strangulata (XP_020201705.1) | MGHHVRRLVLVLLFAAAAMATSEVETTPPIPDQIVLPPLPSPADIPVTPPCLKSISVCALVYQDPSQLAPCCVAVKKLFGSDPECICNGIAEAQKVAKQSGLNYTVDGQEMFRRCEMPLTSCDPEKPGSQSIGNGAPTTRSFGVFQILLVIPLFFMM |
| Hordeum vulgare (KAE8790810.1) | MGHHVRRLVLVLMFATTAMATSEVETTRPIPDQMDLPPLPSPADIPVTPPCLNSISVCGLVYQDPSKLAPCCTAVKKLFGSDPECICDGIAEAQKVAKQSGLNYTVDGQEMFRRCEMPLTSCNPEKPGSQKIGNAAPSARTLGVFQMLLVIPLFFML |
| Triticum aestivum (XP_044430310.1) | MAHHVRRLVLVLLFAAAAMATSEVETTPPIPDQIVLPPLPSPADIPVTPPCLKSISVCALVYQDPSQLAPCCVAVKKLFSSDPECICNGIAEAHKVAKQSGLNYTVDGQEMFRRCEMPLTSCDPEKPGSQSIGNGAPTTRSFGVFQILLVFPLFFMM |
| Triticum aestivum (XP_044378576.1 ) | MGHHVRRLVLVLLFAAAAMATSEVETTPPIPDQIVLPPLPSPADIPVTPPCLKSISVCALVYQDPSQLAPCCVAVKKLFGSDPECICNGIAEAQKVAKQSGLNYTVDGQEMFRRCEMPLTSCDPEKPGSRSIGNGAPTTRSFGVFQILLVIPLFFMM |
| Triticum urartu (XP_048527289.1 ) | MGHHVRRLVLVLLFAAAAMATSEVETTPPIPDQIVLPPLPSPADIPATPPCLNSITVCGAVYGDPSKLAPCCVAVKKLFKSDPECICNAVGEAQKFAKKWGVNDTVDGLEMFRQCQMPTTSCDPRKPGSENIGNAAHTTRSFGGFQILLVFPLFFMM |
| Triticum aestivum (XP_044386271.1) | MGHHVRRLVLVLLFAAAAMATSEVETTPPIPDQIVLPPLPSPADIPATPPCLNSITVCGVVYGDPSKLAPCCVAVKKLFKSDPECICNAVGEAQKFAKKWGVNNTVDGLEMFRQCQMPTTSCDPRKPGSENIGNAAHTTRSFGGFQILLVFPLFFMM |

**Table S3.** Candidate interacting proteins

| ORF | Type and putative protein function |
| --- | --- |
| LOC_Os10g41460 | Transcription factor like protein |
| LOC_Os09g39390 | Oxidoreductase |
| LOC_Os01g15300 | Zinc finger helicase family protein |
| LOC_Os07g37240 | Chlorophyll A-B binding protein |
| LOC_Os02g15230 | GDSL esterase |
| LOC_Os02g47020 | Phosphoribulokinase/Uridine kinase family protein |
| LOC_Os07g47290 | Xylose isomerase |
| LOC_Os12g34980 | Expressed protein |
| LOC_Os06g14510 | Glucose-6-phosphate isomerase |
| LOC_Os02g03260 | 3-isopropylmalate dehydratase large subunit 2 |
| LOC_Os05g49200 | Aspartic proteinase oryzasin-1 precursor |
| LOC_Os08g35740 | 12-oxophytodienoate reductase gene |
| LOC_Os05g43374 | Pseudouridylate synthase |
| LOC_Os08g06630 | Plastid sigma factor |
| LOC_Os02g47020 | Phosphoribulokinase/Uridine kinase family protein |
| LOC_Os05g35170 | NAC transcription factor |
| LOC_Os04g08034 | EMBRYONIC FLOWER2a |
| LOC_Os02g38920 | Glyceraldehyde-3-phosphate dehydrogenase |
| LOC_Os02g42350 | Nitrilase |
| LOC_Os02g30310 | ThiF family domain containing protein |
| LOC_Os03g40270 | UDP-arabinopyranose mutase 1 |
| LOC_Os04g42860 | GDSL-like lipase/acylhydrolase |
| LOC_Os04g52500 | Lecithine cholesterol acyltransferase |
| LOC_Os02g38920 | Glyceraldehyde-3-phosphate dehydrogenase |
| LOC_Os09g36800 | 3-dehydroquinate synthase |
| LOC_Os01g53930 | Hexokinase gene |
| LOC_Os02g27030 | Abiotic stress-induced cysteine proteinase 1 |
| LOC_Os06g36700 | T-complex protein |
| LOC_Os02g41630 | Phenylalanine ammonia-lyase gene |
| LOC_Os06g49470 | Peptidyl-prolyl cis-trans isomerase |
| LOC_Os04g23580 | Xylosyltransferase |
| LOC_Os01g03670 | Dihydroflavonol-4-reductase |
| LOC_Os02g02400 | Catalase |

**Table S4.** Primer information

| Primer name | Sequence (5’-3’), F: Forward; R: Reverse |
| --- | --- |
| RT-*CTB6* | F:CCTCCACCATCTGCGACAA |
|  | R:CGAGTGAGGTGAGGCATTTCTT |
| RT-*CATA* | F:CGTCAACACCTACACCTTCG |
|  | R:CTCGTCGTCCATCAAGCAG |
| RT-*CATB* | F:CCGTCTGGAACAACAACTCC |
|  | R:GGATACGCTCCCTGTCAAAC |
| RT-*CATC* | F:TGCCAAGGAGAACAACTTCA |
|  | R:CCAGTAGGAGAGCCAGATGC |
| RT-*Os02g0802700* | F:CCGAGCCGTTCTTGTTAAGGAT |
|  | R:TCTCCACCTCCCATCAGCAAT |
| RT-*Os03g0719400* | F:CCGAGCCGTTCTTGTTAAGGAT |
|  | R:TCTCCACCTCCCATCAGCAAT |
| RT-*Os12g0563000* | F:TCGTTGGTTTCTCGGGAGGAT |
|  | R:GCCGCCACTTGCTCTTCTC |
| RT-*ACOS12* | F:GAGGGTGGCGTCGTACAAG |
|  | R:TGACGGTTTCATCCTCTTGATG |
| RT-*ABCG15* | F:GCTCGTCCTCATGCTCTTCCT |
|  | R:GACACGTACTTGAGCCACCTTA |
| RT-*ABCG26* | F:CGAAGCGATCGTTCATCAACATG |
|  | R:CGATGCAGACTGTCACAACAATG |
| RT-*PSK10* | F:TCGACGGCTTCATGAGGAACAC |
|  | R:TGCACGCAAGTTCGATAATAGTGG |
| RT-*TDR* | F:GTCAACGTCACCACCTACAAGA |
|  | R:ACCTCGCTGTCCCTCACCAT |
| RT-*TIP2* | F:AACGCCCTCATGCTCCTCATC |
|  | R:TCGCGTCTGAGATCACCGT |
| RT-*PTC1* | F:GTGCGGATGGTCCTGGACAT |
|  | R:CCACCGCCATTGCTGCTC |
| RT-*PTC2* | F:GGCCCGGTCATGGTGATC |
|  | R:TGAGCCCTCCTCTTCTTCCT |
| RT-*Actin* | F:CACAGGTATTGTGTTGGACTCTG |
|  | R:AGTAACCACGCTCCGTCAGG |
| MBP-*CTB6* | F:CGGCCGCGATATCGTCGACGGATCCATGGAACACATCCGCTCTTTC |
|  | R:TTATTTAATTACCTGCAGGGAATTCTCACACTCCCAAAATGAACAG |
| GST-*CATA* | F:GGTTCCGCGTGGATCC ATGGATCCTTGCAAGTTCC |
|  | R:TCGAGTCGACCCGGGAATTCTCACATGCTTGGCTTCAC |
| MBP-*OsC6* | F:CGGCCGCGATATCGTCGACGGATCCATGGCGCCGTCCAAGTCCA |
|  | R:TTATTTAATTACCTGCAGGGAATTCTCAGGCAGATGGAGCCGGG |
| *CATA*-MYC | F:TACACCAAATCGACTCTAGAAAGCTTATGGATCCTTGCAAGTTCC |
|  | R:CAGAAATGAGCTTTTGCTCCATGGTACCCATGCTTGGCTTCACGTTGA |
| *CATA*-GFP | F:TACACCAAATCGACTCTAGAAAGCTTATGGATCCTTGCAAGTTCC |
|  | R:AGCTCCTCGCCCTTGCTCACCATGGTACCCATGCTTGGCTTCACGTTGA |
| *CTB6*-GFP | F:GATACACCAAATCGACTCTAGAAAGCTTATGGCGGCGGGGCTACCGCCG |
|  | R:AGCTCCTCGCCCTTGCTCACCATGGTACCCACTCCCAAAATGAACAG |
| cLUC-*CATA* | F:GCGTCCCGGGGCGGTACCATGGATCCTTGCAAGTTCC |
|  | R:CTGCAGGTCGACTCTAGAGGATCC TCACATGCTTGGCTTCAC |
| *CTB6*-nLUC | F:GAGCTCGGTACCCGGGGATCC ATGGAACACATCCGCTCTTTC |
|  | R:CGCGTACGAGATCTGGTCGAC CACTCCCAAAATGAACAGTAT |
| *CTB6*-YC | F:GGGCTCAGGCCTGGCGCGCCATGGAACACATCCGCTCTTTC |
|  | R:GTACATCCCGGGAGCGGTACCCACTCCCAAAATGAACAGTAT |
| *CATA*-YN | F:GGGCTCAGGCCTGGCGCGCCATGGATCCTTGCAAGTTCC |
|  | R:CTATCGATGGATCCACTAGTCATGCTTGGCTTCACGTTGA |
| *CATA*-AD | F:GTACCAGATTACGCTCATATGATGGATCCTTGCAAGTTCC |
|  | R:ATGCCCACCCGGGTGGAATTC TCACATGCTTGGCTTCAC |
| *CATB-AD* | F:GTACCAGATTACGCTCATATGATGGATCCCTACAAGCATC |
|  | R:ATGCCCACCCGGGTGGAATTC CTACATGTTTGGTTTCAGGT |
| *CATC-AD* | F:GTACCAGATTACGCTCATATGATGGATCCCTACAAGCACCG |
|  | R:ATGCCCACCCGGGTGGAATTC TTACATGCTCGGCTTCGCGC |
| *CTB6*-BD | F:TCAGAGGAGGACCTGCATATGATGGCGGCGGGGCTACCGCCG |
|  | R:TCGACGGATCCCCGGGAATTC TCACACTCCCAAAATGAACAG |
| *OsC6*-AD | F:GTACCAGATTACGCTCATATGATGGCGCCGTCCAAGTCCA |
|  | R:ATGCCCACCCGGGTGGAATTCTCAGGCAGATGGAGCCGGG |
| *CTB6*-Flag (OE) | F:CGTCGACGAGCTCTCTAGAACTAGTATGGAACACATCCGCTCTTTCC |
|  | R:TGATTTTTGCGGAGTACCCGGGTACCTCACACTCCCAAAATGAACAG |
| *ProCTB6*::GUS | F:CCTGTCAAACACTGATAGTTTAAAC TGGTTCGTCATCCGTGTT |
|  | R:CGATCCTCTAGAGTCGAGGCGCGCC GATTGCTGTATGTCCGAGGG |
| pESC-*CTB6* (Flag) | F:AAGAATTTTTGAAAATTCGAATTCATGGAACACATCCGCTCTTTC |
|  | R:CCCTTTAGTGAGGGTTGAATTCCACTCCCAAAATGAACAGTAT |
| pESC-*CATA* (Myc) | F:ACTATAGGGCCCGGGCGTCGACATGGATCCTTGCAAGTTCC |
|  | R:TCAACTTCTGTTCCATGTCGACCATGCTTGGCTTCACGTT |
| pESC-*CATB* (Myc) | F:ACTATAGGGCCCGGGCGTCGACATGGATCCCTACAAGCATC |
|  | R:TCAACTTCTGTTCCATGTCGACCATGTTTGGTTTCAGGTTGA |
| pESC-*CATC* (Myc) | F:ACTATAGGGCCCGGGCGTCGACATGGATCCCTACAAGCACCG |
|  | R:TCAACTTCTGTTCCATGTCGACCATGCTCGGCTTCGCGCTGA |
| Antisense probe by in situ hybridization | GCCAGTGCTGCTAGGAGGAAAGAGCGGATGTG |

**Table S5.** The relative seed-setting rate of rice accessions

| Accession NO. | Relative seed-setting rate | Hap. | Accession NO. | Relative seed-setting rate | Hap. |
| --- | --- | --- | --- | --- | --- |
| CH1122 | 0.96 | Hap1-K | CH1017 | 0.57 | Hap3 (Sub-I) |
| CH1163 | 0.71 |  | CH1019 | 0.59 |  |
| CH1180 | 0.85 |  | CH1022 | 0.72 |  |
| CH1097 | 0.64 |  | CH1044 | 0.86 |  |
| CH1179 | 0.75 |  | CH1056 | 0.27 |  |
| CH1196 | 0.76 |  | CH1100 | 0.70 |  |
| CH1199 | 0.72 |  | CH1129 | 0.60 |  |
| LJ2 | 0.80 |  | CH1137 | 0.61 |  |
| LJXTHG | 0.72 |  | CH1145 | 0.51 |  |
| KMXBG | 0.87 |  | CH1149 | 0.59 |  |
| LJXHG | 0.82 |  | CH1158 | 0.31 |  |
| CT | 0.85 |  | CH1159 | 0.19 |  |
| CH1305 | 0.37 | Hap2-T | CH1164 | 0.70 |  |
| CH1009 | 0.77 |  | CH1165 | 0.73 |  |
| CH1070 | 0.72 |  | CH1166 | 0.49 |  |
| CH1274 | 0.67 |  | CH1170 | 0.80 |  |
| CH1302 | 0.80 |  | CH1204 | 0.79 |  |
| CH1296 | 0.50 |  | CH1208 | 0.25 |  |
| CH1003 | 0.34 |  | CH1209 | 0.74 |  |
| CH1008 | 0.68 |  | CH1210 | 0.69 |  |
| CH1071 | 0.41 |  | CH1214 | 0.84 |  |
| CH1002 | 0.78 |  | CH1215 | 0.53 |  |
| CH1010 | 0.81 |  | CH1227 | 0.65 |  |
| CH1069 | 0.81 |  | CH1229 | 0.67 |  |
| CH1125 | 0.71 |  | CH1230 | 0.65 |  |
| CH1266 | 0.11 |  | CH1234 | 0.69 |  |
| CH1281 | 0.75 |  | CH1245 | 0.24 |  |
| CH1290 | 0.74 |  | CH1246 | 0.80 |  |
| CH1249 | 0.52 |  | CH1255 | 0.55 |  |
| CH1005 | 0.68 | Hap3 (Sub-J) | CH1265 | 0.82 |  |
| CH1021 | 0.82 |  | CH1267 | 0.78 |  |
| CH1059 | 0.77 |  | CH1276 | 0.73 |  |
| CH1060 | 0.67 |  | CH1277 | 0.83 |  |
| CH1091 | 0.19 |  | CH1278 | 0.35 |  |
| CH1092 | 0.63 |  | CH1293 | 0.74 |  |
| CH1223 | 0.74 |  | CH1057 | 0.19 |  |
| CH1250 | 0.60 |  | CH1064 | 0.64 |  |
| CH1006 | 0.78 |  | CH1106 | 0.52 |  |
| CH1020 | 0.84 |  | CH1107 | 0.68 |  |
| CH1083 | 0.71 |  | CH1133 | 0.58 |  |
| CH1088 | 0.62 | Hap3 (Sub-J) | CH1146 | 0.68 | Hap3 (Sub-I) |
| CH1105 | 0.34 |  | CH1152 | 0.87 |  |
| CH1034 | 0.67 | Hap4 | CH1171 | 0.82 |  |
| CH1063 | 0.80 |  | CH1173 | 0.50 |  |
| CH1082 | 0.40 |  | CH1181 | 0.66 |  |
| CH1087 | 0.65 |  | CH1220 | 0.54 |  |
| CH1126 | 0.48 |  | CH1262 | 0.67 |  |
| CH1141 | 0.51 |  | CH1263 | 0.78 |  |
| CH1155 | 0.56 |  | CH1271 | 0.06 |  |
| CH1242 | 0.71 |  | CH1272 | 0.83 |  |
| CH1269 | 0.47 |  | CH1273 | 0.74 |  |
| CH1284 | 0.84 |  | CH1282 | 0.49 |  |
| CH1147 | 0.84 |  | CH1285 | 0.08 |  |
| CH1156 | 0.59 |  | CH1286 | 0.44 |  |
| CH1244 | 0.59 |  |  |  |  |
| CH1275 | 0.51 |  |  |  |  |
| CH1051 | 0.20 |  |  |  |  |
